# The composition and functional protein subsystems of the human nasal microbiome in granulomatosis with polyangiitis: a pilot study

**DOI:** 10.1186/s40168-019-0753-z

**Published:** 2019-10-22

**Authors:** Josef Wagner, Ewan M. Harrison, Marcos Martinez Del Pero, Beth Blane, Gert Mayer, Johannes Leierer, Seerapani Gopaluni, Mark A. Holmes, Julian Parkhill, Sharon J. Peacock, David R. W. Jayne, Andreas Kronbichler

**Affiliations:** 1 Wellcome Sanger Institute, Wellcome Genome Campus, Hinxton, Cambridgeshire CB10 1SA UK; 2 Department of Medicine, University of Cambridge, Box 157, Addenbrooke’s Hospital, Hills Road, Cambridge, CB2 0QQ UK; 3 Department of Public Health and Primary Care, University of Cambridge, Strangeways Research Laboratory, Worts Causeway, Cambridge, CB1 8RN UK; 4 West Suffolk Hospital, Hardwick Lane, Bury St. Edmunds, Suffolk UK; 5 Department of Internal Medicine IV (Nephrology and Hypertension), Medical University Innsbruck, Innsbruck, Austria; 6 Vasculitis and Lupus Clinic, Box 57, Addenbrooke’s Hospital, Hills Road, Cambridge, CB2 0QQ UK; 7 Department of Veterinary Medicine, University of Cambridge, Cambridge, UK; 8 London School of Hygiene and Tropical Medicine, WC1E 7HT, London, UK

**Keywords:** GPA, Microbiome, ANCA, Vasculitis, *Staphylococcus*, rRNA sequencing, Metagenomics

## Abstract

**Background:**

Ear, nose and throat involvement in granulomatosis with polyangiitis (GPA) is frequently the initial disease manifestation. Previous investigations have observed a higher prevalence of *Staphylococcus aureus* in patients with GPA, and chronic nasal carriage has been linked with an increased risk of disease relapse. In this cross-sectional study, we investigated changes in the nasal microbiota including a detailed analysis of *Staphylococcus* spp. by shotgun metagenomics in patients with active and inactive granulomatosis with polyangiitis (GPA). Shotgun metagenomic sequence data were also used to identify protein-encoding genes within the SEED database, and the abundance of proteins then correlated with the presence of bacterial species on an annotated heatmap.

**Results:**

The presence of *S. aureus* in the nose as assessed by culture was more frequently detected in patients with active GPA (66.7%) compared with inactive GPA (34.1%). Beta diversity analysis of nasal microbiota by bacterial 16S rRNA profiling revealed a different composition between GPA patients and healthy controls (*P* = 0.039). Beta diversity analysis of shotgun metagenomic sequence data for *Staphylococcus* spp. revealed a different composition between active GPA patients and healthy controls and disease controls (*P* = 0.0007 and *P* = 0.0023, respectively), and between healthy controls and inactive GPA patients and household controls (*P* = 0.0168 and *P* = 0.0168, respectively). Patients with active GPA had a higher abundance of *S. aureus*, mirroring the culture data, while healthy controls had a higher abundance of *S. epidermidis*. *Staphylococcus pseudintermedius*, generally assumed to be a pathogen of cats and dogs, showed an abundance of 13% among the *Staphylococcus* spp. in our cohort. During long-term follow-up of patients with inactive GPA at baseline, a higher *S. aureus* abundance was not associated with an increased relapse risk. Functional analyses identified ten SEED protein subsystems that differed between the groups. Most significant associations were related to chorismate synthesis and involved in the vitamin B_12_ pathway.

**Conclusion:**

Our data revealed a distinct dysbiosis of the nasal microbiota in GPA patients compared with disease and healthy controls. Metagenomic sequencing demonstrated that this dysbiosis in active GPA patients is manifested by increased abundance of *S. aureus* and a depletion of *S. epidermidis*, further demonstrating the antagonist relationships between these species. SEED functional protein subsystem analysis identified an association between the unique bacterial nasal microbiota clusters seen mainly in GPA patients and an elevated abundance of genes associated with chorismate synthesis and vitamin B_12_ pathways. Further studies are required to further elucidate the relationship between the biosynthesis genes and the associated bacterial species.

## Background

Granulomatosis with polyangiitis (GPA, formerly Wegener’s granulomatosis) is a multi-system autoimmune disorder. Disease aetiopathogenesis is considered to be multi-factorial but includes a host genetic component, epigenetic modifications and the environment [1, 2], with an increased risk of developing GPA in farmers or those with a high occupational solvent exposure in the index year or during their working lifetime [3]. Other studies have demonstrated an association with dust exposure, and a correlation between lifetime exposure to high doses of silica and anti-neutrophil cytoplasm antibody (ANCA) positivity [4, 5].

Patients with GPA have a higher rate of nasal colonisation by *Staphylococcus aureus* (60–70%) than the general population (20–30%), and the presence of persistent carriage has been associated with an increased risk of disease relapse during follow-up [6, 7]. A randomised controlled trial showed a reduction of relapses following daily administration of trimethoprim-sulfamethoxazole (TMP-SMX) administered over a 2 -year period [8]. These findings suggest that GPA patients have a perturbed nasal microbiota, which may be related or contribute to the high *S. aureus* colonisation rate.

In this study, we aimed to investigate the nasal microbiota in GPA patients by microbiome analysis of nasal swabs obtained from GPA patients in an active and inactive disease state and controls (disease controls, healthy household controls and healthy hospital personnel). In addition, shotgun metagenomic sequences were used to identify differences in functional SEED protein subsystems between the sample groups and their association with the most abundant species.

## Results

### Cohort and sampling

A case-control study was conducted, including 12 active GPA patients (aGPA), 44 inactive GPA patients (inGPA), and 13 disease controls (DC) (three with microscopic polyangiitis and 10 with eosinophilic GPA). The healthy control group comprised four healthy household controls (HHC) related to patients with aGPA (spouse or partner) and 11 unrelated healthy controls (HC) (hospital employees). The average age across all 84 participants was 55.4 years (17–87). Detailed clinical data for the patients and controls is given in Table 1. A total of 97 nasal swabs were collected from 56 patients with a history of active ear, nose and throat (ENT) involvement (12 being active during sampling), from 13 DC and from 15 healthy controls. Follow-up swabs were obtained from 12 patients to investigate temporal changes, including five swabs of patients initially classified as aGPA after remission was achieved a month later. The residual samples were obtained from patients with inGPA and one HHC.

**Table 1:** Clinical characteristic of patients and controls

Participant characteristics	Total subjects	Active GPA (aGPA)	Inactive GPA (inGPA)^a^	Disease controls: microscopic polyangiitis (MPA) and eosinophilic GPA (EGPA)	Healthy controls (HC)	Healthy household controls (HHC)
	84	12	44	13	11	4
Age (years)	57.9 (17–73)	56.1 (42–73)	58.2 (17–87)	58.4 (42–76)	39.4 (23–58)	
Gender female	46.30%	50%	47.60%	38.50%	81.80%	50%
Gender male	53.70%	50%	52.40%	61.50%	18.20%	50%
Disease duration (months)	113.3	78	124.7	110.8		
BVAS WG	1.1	3.5	0.7	0.2		
VDI	2.0	2.1	1.5	3.5		
DEI	1.5	3.7	1.2	0.4		
Steroids	56.7%	75%	47.6%	69.2%		
Immunosuppression^b^	59.7%	75%	57.1%	53.8%		
CRP (mg/dl)	7.6	14.2	7.4	2.2		
ESR (mm/h)	13.4	24.1	11	10.3		
eGFR (MDRD)	87.2	83.8	87.3	90.3		
Epistaxis	19.4%	83.3%	4.8%	7.7%		
Mucopurulent discharge	23.9%	100%	9.5%	0%		
Olfaction	19.4%	50%	7.1%	30.8%		
Crusting	37.3%	75%	38.1%	0%		
Inflammation	24.2%	100%	10.5%	0%		
Bloody discharge	16.1%	90.9%	0%	0%		
Nasal obstruction	16.1%	63.6%	7.9%	0%		

*Abbreviations*: *BVAS WG* Birmingham Vasculitis Activity Score for Wegener’s Granulomatosis (WG, GPA), *VDI* Vasculitis Damage Index, *DEI* Disease Extent Index, *CRP* C-reactive protein, *ESR* erythrocyte sedimentation rate, *eGFR* estimated glomerular filtration rate, *MDRD* Modification of Diet in Renal Disease

^a^Inactive ear, nose and throat (ENT) GPA

^b^Within the year of sample collection

### Staphylococcus spp. culture

Bilateral nasal swabs were taken and plated onto culture media that was selective for *S. aureus*. Twenty-nine subjects (34.5%) were positive for *S. aureus* (aGPA 8/12 [66.7%], inGPA 15/44 [34.1%], DC 3/13 [23.1%], HC 2/11 [18.2%] and HHC 1/4 [25%]).

We next sought to investigate the *S. aureus* isolates by antimicrobial susceptibly testing, since a high frequency of TMP-SMX and ciprofloxacin resistance in *S. aureus* obtained from GPA patients was recently reported [9]. None of the *S. aureus* isolates were methicillin resistant (MRSA), and three isolates were completely susceptible to all antibiotics tested (Additional file 6: Table S1). Phenotypic resistance to benzylpenicillin (*n* = 22, 75.9%), erythromycin (*n* = 7, 24.1%) and mupirocin (*n* = 4, 13.8%) was common, with small numbers of isolates exhibiting resistance to ciprofloxacin (*n* = 1, 3.4%), fusidic acid (*n* = 2, 6.9%), tetracycline (*n* = 2, 6.9%) and trimethoprim (*n* = 1, 3.4%) (Additional file 6: Table S1).

To further investigate the *S. aureus* isolates, we subjected the 32 isolates (8 aGPA, 15 inGPA, 1 HHC, 2 HC and 3 DC, 3 longitudinal samples) to whole genome sequencing (WGS) (Table 2). Elucidation of multilocus sequence types (MLST) from the WGS data identified that there were 18 unique sequence types (STs) (Additional file 7: Table S2) with only three STs being found in more than one GPA patients, namely ST45 (*n* = 4), ST15 (*n* = 3) and ST398 (*n* = 2). Generation of core genome-based phylogenies revealed that in both GPA patients with sequential samples the same closely related strain was present at both time points (patients 63 (ST425) and 21 (ST398) (Additional file 1: Fig. S1). Individual phylogenies for each of the three STs (ST398, ST45 and ST15) revealed that all isolates from the GPA patients were distantly related (> 100 single nucleotide polymorphisms SNPs—*S. aureus* isolates that are < 50 SNPs apart are regarded as suggestive of recent transmission) [10], suggesting that none of these clusters were recent transmission events between GPA patients. The only likely transmission event was between GPA patient 21 and their HHC partner (P23 in Additional file 1: Fig. 1a), since their two pairs of isolates only differed by ~ 20 SNP. Analysis of the genome content revealed that four isolates, from four different STs (STs 30, 34, 39 and 3804) were positive for the toxic shock toxin gene (*tst*), which has been implicated previously in GPA relapse [11]. Three of these were in active GPA disease (patients 33, 51 and 125) at the time of sampling, and the fourth was from an inactive GPA case (patient 121), who subsequently relapsed 11 months later. None of the HC or DC were positive for a *tst* positive isolate (Additional file 7: Table S2).

**Table 2:** Patient samples used for sequencing and MALDI TOF

Samples	Total	aGPA	inGPA	DC	HC	HHC	Longitudinal
V1 V2 16S^a^	59	7	31	2	7	4 (3 patients)	8 (7 patients)
Shotgun sequenced	96	12	43	13	11	5 (4 patients)	12 (11 patients)
MALDI-TOF	83	10	36	12	0	3	9
Cultured *S. aureus*	32	8	15	3	2	1	2
Cultured *S. warneri*	15	1	4	5	2	1	2
Cultured *S. pasteuri*	3	0	3	0	0	0	0
Cultured *S. capitis*	11	1	5	3	2	0	0
Cultured *S. epidermidis*	13	0	7	1	3	1	1
Cultured *S. pseudintermedius*	4	0	2	0	0	0	2 (1 patient)
Cultured *S. haemolyticus*	17	0	8	4	2	1	2
Cultured *S. caprae*	1	1	0	0	0	0	0
Cultured *S. saphrophyticus*	1	0	1	0	0	0	0
Cultured *S. lugdunensis*	2	0	1	0	1	0	0
Cultured *S. simulans*	1	0	0	1	0	0	0
Cultured *S. pettenkoferi*	1	0	0	0	1	0	0

*Abbreviations*: *aGPA* active granulomatosis with polyangiitis, *inGPA* inactive GPA, *DC* disease controls (microscopic polyangiitis and eosinophilic GPA), *HC* healthy controls, *HHC* healthy household controls, *MALDI-TOF* matrix-assisted laser desorption/ionisation–time-of-flight mass spectrometry

^a^For the bacterial 16S rRNA sequencing project, we sequenced the bacterial 16S rRNA variable region v1 and v2

**Fig. 1:**
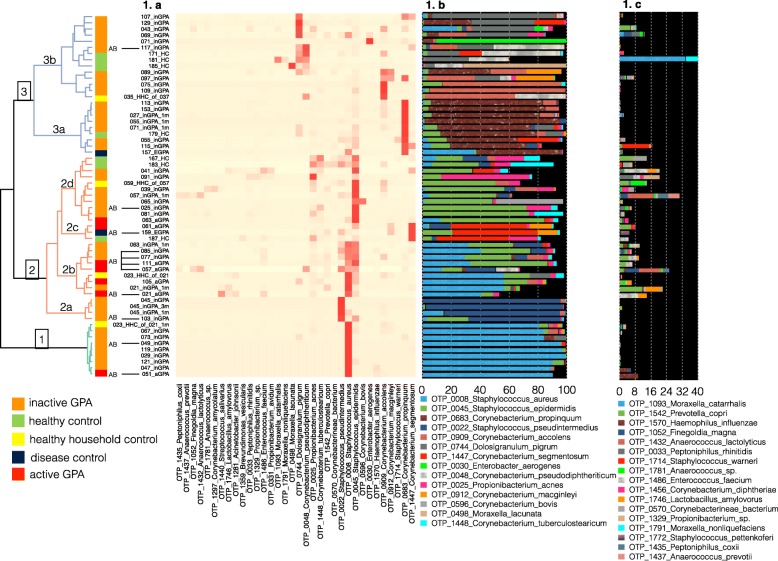
Hierarchical clustering and taxonomic annotation of bacterial 16S rRNA marker gene sequenced species. Bacterial 16S sequence data were available from 59 samples including seven active GPA, 31 inactive GPA, two disease controls (EGPA), seven unrelated healthy controls, four healthy household controls, and eight longitudinal samples. **a** Hierarchical clustering with heatmap presentation was done with R package Heatplus (v 2.20.0, Author: Alexander Ploner). For the heatmap presentation, we removed species with less than 5 % as their maximum relative abundance in five samples which resulted in the inclusion of 34 oligotype species. **b** A stacked bar chart showing the distribution of the top 14 species (minimum abundance of 1% covering 93.16% of all reads) is placed next to the heatmap. **c** A stacked bar chart showing the distribution of the next top 17 species (minimum abundance between 0.1% and 1% covering 5.56% of all reads)

### Bacterial 16S rRNA marker gene analysis

Next, we investigated the composition of the nasal microbiota in GPA patients in comparison to DC and HC. Bacterial 16S sequence data was generated for 59 samples (7 aGPA, 31 inGPA, 2 DC, 7 HC, 4 HHC and 8 longitudinal including aGPA, inGPA and HHC cases). The 16S sequences were used for oligotyping, which generates closely related bacterial clusters called oligotypes. A total of 64 oligotype (OTP) species were identified which were then used for heatmap analysis and calculation of species abundance in the individual samples (Fig. 1). Hierarchical clustering as part of the heatmap analysis identified three main clusters based on dominant species, with clusters 2 and 3 both having distinct sub-clusters (Fig. 1a). Cluster 1 was made up of samples from GPA patients and one HHC, which was dominated by *S. aureus*. Cluster 2 contained samples from all five patient groups and was dominated by *S. aureus and S. epidermidis*. However, individual sub-clusters were dominated by other species, such as cluster 2.a, which was dominated by *S. pseudintermedius*, though three of four samples were from a single inGPA patient (patient 45). Cluster 3 did not contain active GPA patients and was characterised by two sub clusters; cluster 3.a was dominated by *Corynebacterium propinquum*, and cluster 3.b was more diverse and dominated by *Dolosigranulum pigrum*, *Corynebacterium pseudodiphtheriticum*, *Corynebacterium accolens* and *Enterobacter aerogenes* in one case. Notably, *S. aureus* and *S. epidermidis* were the least common species in cluster 3. Samples from patients on antibiotic treatment (denoted AB in Fig. 1) were detected in all clusters.

We then examined inter-individual variability in microbial profiles of first time point samples using a non-metric multidimensional scaling (NMDS) plot (Additional file 2: Figure S2**)**. NMDS represents the original position of data (samples) in multidimensional space as accurately as possible using a reduced number of dimensions that can be easily plotted and visualised. NMDS revealed that the microbiome in the samples from HC group clustered furthest away from the aGPA and inGPA patients (Additional file 2: Figure S2a). Statistical testing of this clustering using a permutational analysis of variance (PERMANOVA) test revealed that the microbiome composition (beta diversity) between the five groups did not differ (*P* > 0.05). However, when GPA patients (aGPA and inGPA) were grouped together (GPA in Additional file 1: Figure S2b), the microbiome cluster was statistically different to the HC cluster (PERMANOVA test: *P* = 0.039, *F* = 1.739), demonstrating that patients with GPA had a distinct nasal microbiota compared with HC. We next questioned whether patients in the inGPA group with a high relative *S. aureus* abundance had a higher relapse rate during follow-up. In the 16S sequenced group, patients with a relapsing disease course (*n* = 16) had a relative mean abundance of 30.14, while those with a non-relapsing course (*n* = 15) had a mean abundance of 22.82 (see Additional file 8: Table S3). Unpaired *t* test revealed no statistical association between *S. aureus* mean abundance and disease outcome (*P* value, unpaired *t* test = 0.5739).

We further tested whether any of the top 1% species presented in Fig. 1 showed an association with any of the five sample groups. None of the top 1% species were statistically different in any of the five sample groups.

To further understand the perturbations in the nasal microbiota of GPA, we examined longitudinal changes in the nasal microbiome in a subset of six patients for whom we had time course samples, together with HHC in two cases (Additional file 3: Figure S3). Bacterial 16S rRNA gene profiles were clearly distinct between the individual case studies, while the individual cases retained a broadly consistent profile at the different time points. The two HHC displayed a similar microbiome profile to their GPA household partner, though with some obvious differences.

### Shotgun sequence analysis of the nasal microbiome

We further investigated the nasal microbiome using shotgun metagenomic sequencing. This aimed to acquire deep sequence information in addition to bacterial 16S sequences. Ninety-six samples across all recruited patients and controls were available (Table 2). MEGAN identified unique hits to a total of 424 bacterial taxa with a minimum hit abundance of 0.01% which covers 96.4% of all MEGAN hits. The *Staphylococcus* taxa made up 20.4% of all MEGAN hits. We focused on the retrieved *Staphylococcus* taxa since it was the most abundant taxa in the shotgun metagenomic sequences and is commonly reported to be implicated in GPA. Importantly, we have previously shown that metagenomic analysis of low-biomass samples is plagued by reagent contamination [12]. Crucially, *Staphylococcus* taxa provided reliable results without evidence of contamination in the contamination controls. A total of 198 different *Staphylococcus* hits were extracted from the shotgun sequence analysis with a minimum abundance of 0.001% and a maximum abundance of 32.1%. Seven *Staphylococcus* species were in the minimum 1% abundance group which covered 88.61% of all *Staphylococcus* hits in MEGAN. Thirty-two species were in the minimum 0.1% abundance group, which covered 97.49% of all *Staphylococcus* hits in MEGAN (Additional file 9: Table S4).

**Fig. 2:**
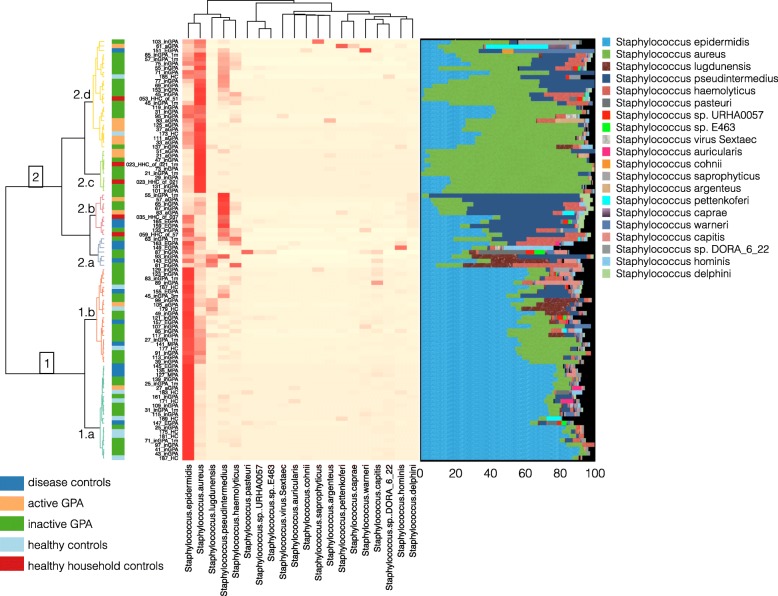
Hierarchical clustering and taxonomic annotation of shotgun-sequenced *Staphylococcus* species. For the heatmap analysis, we removed species with less than 5% as their maximum relative abundance in five samples, which retained 20 species for easier presentation in the heatmap. The same 20 species were used for the stacked bar chart. Left over black bars represent other species not present in the top 20 species

Shotgun-sequenced *Staphylococcus* species were analysed in the same way as bacterial 16S sequence data. For easier presentation of the heatmap, we removed species with less than 5% as their maximum relative abundance in less than five samples leaving top 20 species (Fig. 2). The hierarchical clustering (grouping of *Staphylococcus* species) seen in the heatmap revealed two main clusters. Cluster one (all sample groups except HHC samples) was dominated by *S. epidermidis* together with a small proportion of *S. aureus* in sub cluster 1a and a larger proportion of *S. aureus* in sub cluster 1b. We subdivided cluster two into four sub-clusters, whereby cluster 2a was the most diverse cluster with six samples. Cluster 2b was dominated by *S. pseudintermedius*. No HC samples were found in clusters 2a and 2b. Cluster 2c was dominated by *S. aureus* and cluster 2d was dominated by *S. aureus*, *S. epidermidis* and *S. pseudintermedius*. Cluster 2d contained the largest proportion of aGPA patients.

**Figure 3:**
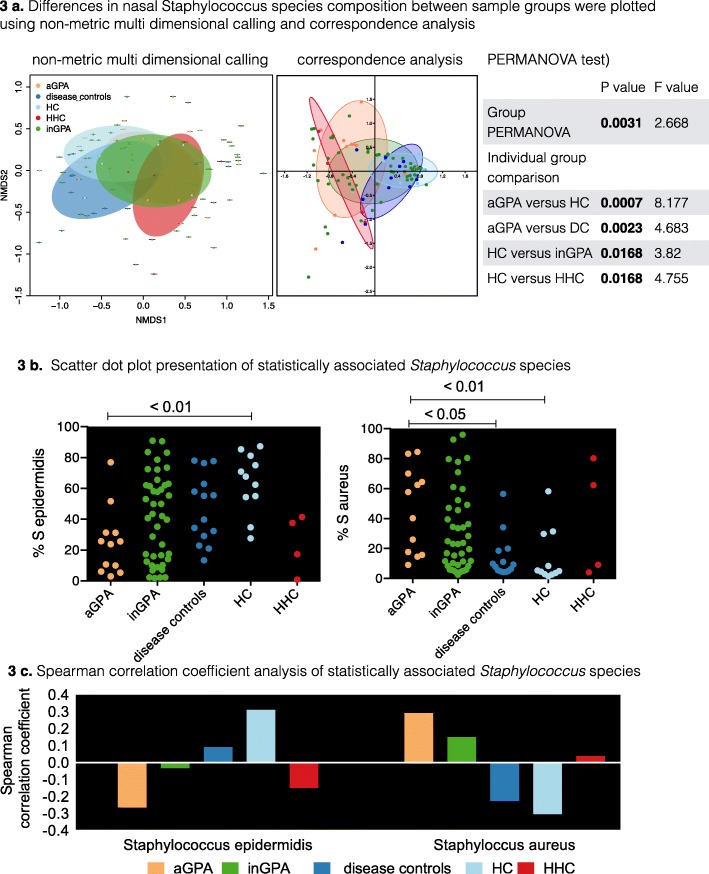
Differences in nasal *Staphylococcus* species composition between sample groups. **a** Differences in shotgun-sequenced nasal *Staphylococcus* species composition between sample groups were visualised using non-metric multi-dimensional scaling (NMDS plot) and correspondence analysis (CA plot). The significance of the separation between the different sample groups was further assessed by PERMANOVA test (statistical test for bacterial beta diversity). The overall group comparison was statistically different (*P* = 0.0031). The individual group comparisons revealed statistical differences in beta diversity between aGPA patients and HC (*P* = 0.0007) and between aGPA patients and disease controls (*P* = 0.0023). Beta diversity was also statistically different between the HC and inGPA patients (*P* = 0.0168) and between HC and HHC (*P* = 0.0168). **b** Scatter dot plot presentation of statistically associated *S. epidermidis* and *S. aureus*. *S. epidermidis* was found at statistically higher abundance in the HC group compared with aGPA patients. *S. aureus* was found at statistically higher abundance in aGPA patients compare to DC patients and the HC groups. **c** The direction of the Spearman’s correlation coefficient value (positive or negative value on the *y*-axis) determines whether *S. epidermidis* and *S. aureus* are either positively or negatively associated with the different sample groups. aGPA, active granulomatosis with polyangiitis (GPA); inGPA, inactive GPA; DC, disease controls (eosinophilic GPA and microscopic polyangiitis); HC, unrelated healthy controls; HHC, healthy household controls; PERMANOVA, permutational multivariate analysis of variance

We examined inter-individual variability in *Staphylococcus* spp. profiles using NMDS and correspondence analysis (CA) (Fig. 3a). NMDS and CA together with PERMANOVA testing revealed that the overall group difference was different (PERMANOVA test: *P* = 0.0031, *F* = 2.668). Individual group comparison revealed that the aGPA patients were different to the HC (*P* = 0.0007, *F* = 8.177) and DC (*P* = 0.0023, *F* = 4.683). In addition, the HC were different to the inGPA patients (*P* = 0.0168, *F* = 3.82) and HHC (*P* = 0.0168, *F* = 4.755). NMDS and CA indicated that the DC was similar to HC compared with the GPA patients. The top seven nasal *Staphylococcus* species which were in the top 1% abundance group (Additional file 9: Table S4) were further analysed in detail using scatter dot plot presentation together with a Kruskal-Wallis test. *S. epidermidis* (32.9% abundance) was detected at statistically higher abundance in HC compared with aGPA patients (Fig. 3b). In contrast, *S. aureus* (29.71% abundance) was detected at statistically higher abundance in aGPA patients compared with DC and HC but was not different to inGPA patients (Fig. 3b). Figure 3c demonstrates the direction of association of *S. epidermidis* and *S. aureus* in the five sample groups. In line with results obtained from 16S rRNA analysis, no association with relapsing disease course in those with a high *S. aureus* abundance (*P* value, Mann-Whitney test = 0.3581) was observed.

**Fig. 4:**
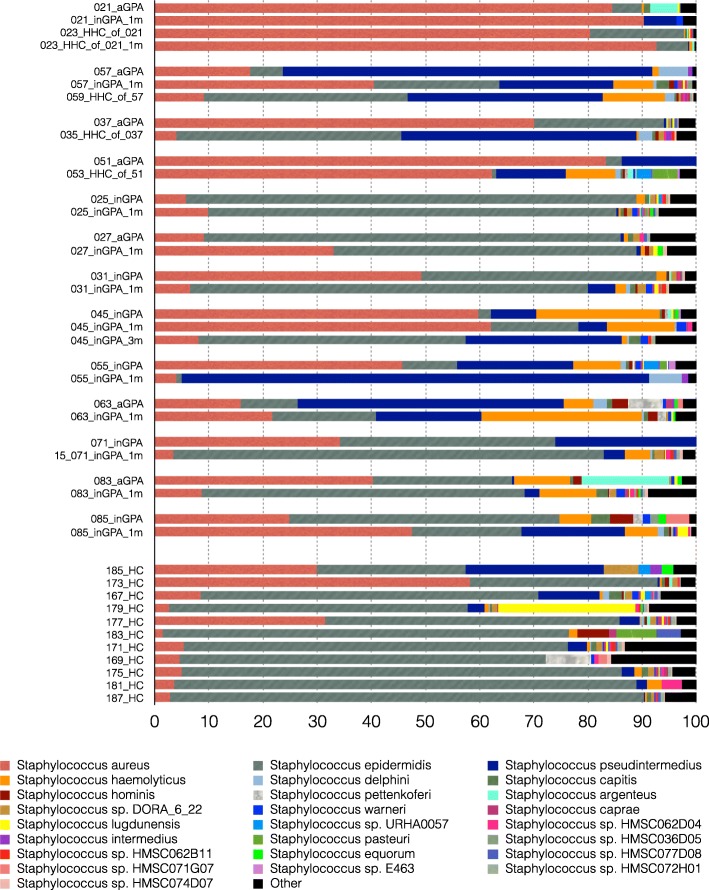
Taxonomic annotation of longitudinal case studies of shotgun-sequenced *Staphylococcus* species. Shotgun-sequenced *Staphylococcus* species were analysed in 13 longitudinal case studies together with healthy controls. The individual case studies were grouped together with follow-up samples 1 month and 3 months later (where available) and with or without healthy household controls at the time of initial sampling and 1 month later for one case study. The *x*-axis shows the proportional abundance of the top 25 species with a minimum abundance of 0.1% across the patient samples, which covers 97.85% of all *Staphylococcus* reads within the longitudinal cohort. The matching species of the healthy controls are presented at the bottom of Additional file 4: Figure S4 for comparisons. aGPA, active granulomatosis with polyangiitis (GPA); inGPA, inactive GPA; HC, unrelated healthy controls, HHC, healthy household controls

We examined longitudinal changes in the nasal *Staphylococcus* profile in a subset of 13 patients with sequential samples. Figure 4 demonstrates that the overall composition of *Staphylococcus* species from the initial samples remained similar over time. However, the relative proportion of species changed in some participants (e.g. 057_aGPA or 031_inGPA), while in other participants, it remained similar (e.g. 021_aGPA or 025_inGPA). Overall, HHC samples showed similar *Staphylococcus* composition compared with their GPA-affected spouses/partners.

### Metagenomic functional profiling

We next sought to gain further insights into the nasal microbiome dysbiosis of patients with GPA by performing functional profiling of the metagenomic data, in order to identify genes and pathways that were significantly different between the groups. For the functional profiling, we used the SEED protein database analysis in MEGAN. At level 1 SEED classification, a total of 43 subsystems were identified which were considered too shallow, and hence we conducted a level two classification. SEED classification has three levels, and level one classification is the most basic classification comparable to phylum level speciation in bacteria. The proteins annotated at level two classification identified 971 subsystems. Out of the 971 subsystems, 319 had a minimum abundance of 0.1% across all samples which covered 82.52% of all SEED hits in MEGAN. These 319 subsystems were used for downstream statistical analysis using the non-parametric Kruskal-Wallis test. The 319 SEED classifications are shown in Additional file 10: Table S5.

**Fig. 5:**
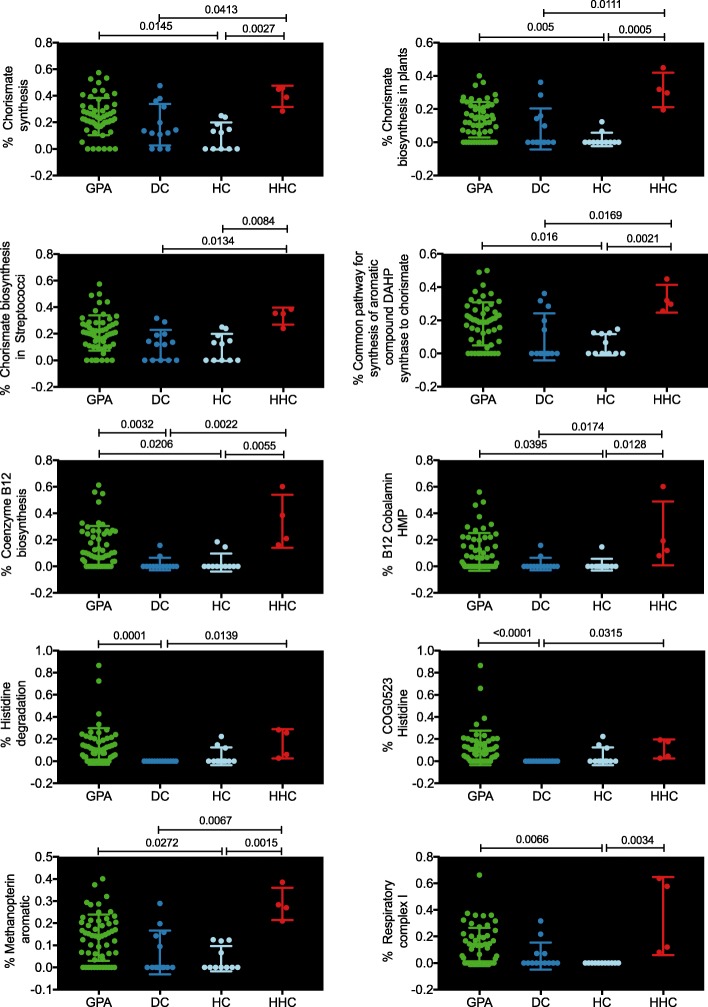
Statistically significant functional SEED annotation pathway. Shotgun sequences were used for the analysis of SEED functional protein subsystems. Ten SEED functional protein subsystems were statistically associated with the four sample groups and are shown in Fig. 5. GPA, granulomatosis with polyangiitis (GPA), DC, disease controls; HC, healthy controls; HHC, healthy household controls

Ten SEED functions were identified to be statistically different within the four groups. These ten functions had a Kruskal-Wallis test FDR-corrected *P* value between 0.0046 and 0.0432. Dunn’s multiple comparison test revealed that these ten functions were differently associated within the four groups (Fig. 5). For the SEED functional analysis, we grouped the active GPA samples and the inactive GPA samples into one group called “GPA” due to similarities in their functional analyses (data not shown). Overall, the HC group showed the lowest abundance of these ten SEED subsystems, and in most cases, the HHC had a statistically significant higher abundance of the same subsystems compared with the DC and/or the HC group. The GPA patients were significantly enriched for genes in 7/10 SEED subsystems compared with the HC group.

**Fig. 6:**
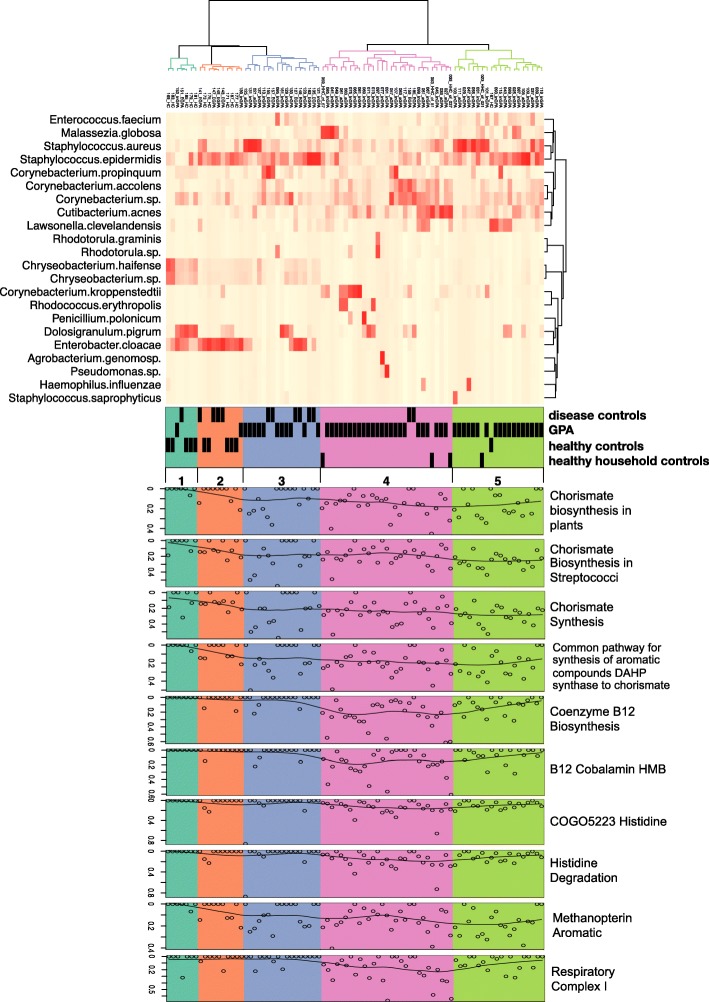
Correlation between metagenomic species and SEED functional protein subsystems. Most abundant shotgun metagenomic species were correlated with the ten statistically associated SEED functional protein subsystems. The cuth parameter in the dendrogram was set in such a way that it identified five clusters which are colour coded. The cuth parameter sets the height at which to cut through the dendrogram to define groups of similar features/samples. A distance metric was generated with R function “vegist” from the VEGAN package using the “bray” method and Hclust R function from the VEGAN package using the ward. D method was used to cluster the distance matrix. The heatmap was generated with the Heatplus package from R, version 2.26.0

We next combined the ten SEED subsystems and shotgun species abundance into an annotated heatmap that provided some insights into the correlation between the species and SEED subsystems (Fig. 6). Clusters 1 and 2 contain the majority of the healthy control (10/11) and is dominated by *Staphylococcus epidermidis*, *Dolosigranulum pigrum*, *Enterobacter cloacae*, and to a lesser extent by two *Chryseobacterium* species. Cluster 3 contained nearly half of the DC samples (6/13 and several GPA samples) and was dominated by *Staphylococcus epidermidis* and in few samples by *Dolosigranulum pigrum and Enterobacter cloacae*. Clusters 4 and 5 contained most of the GPA patients samples and all four of the matched HHC. Multiple *Corynebacterium* species, *Cutibacterium acnes*, and *S. aureus* and *S. epidermidis* (mainly cluster 5) were found in clusters 4 and 5. Elevated SEED functional pathways were detected mostly in clusters 3, 4 and 5. Elevated SEED functional pathways in cluster 3 were dominated by genes involved in the chorismate and methanopterin aromatic function. In contrast, clusters 4 and 5 were particularly enriched for genes involved in vitamin B_12_ and chorismate synthesis. The HC in clusters 1 and 2 together with DC samples in the clusters 1, 2 and 3 showed the least elevated abundance of the SEED functional genes.

### The impact of disease duration on changes in the microbiome

Next, we investigated the association between the duration of the disease for each patient (the time since first diagnosis of GPA until study enrolment) and dysbiosis of the microbiome. For this purpose, we analysed the bacterial 16S dataset and *Staphylococcus* shotgun-sequenced dataset using an unbiased approach based on hierarchical clustering and heatmap analysis with annotation for disease duration in months and by patient groups. For the bacterial 16S dataset, we generated three clusters (coloured green, orange, and blue) in the heatmap (Additional file 4: Figure S4). Analysis of beta diversity using a PERMANOVA test revealed that the microbiome composition between the green, orange and blue clusters identified in the heatmap (Additional file 4: Figure S4) was statistically different to each other (*P* = 0.003, Bonferroni-corrected *P* value).

Non-parametric Kruskal-Wallis test with Dunn’s multiple comparison test using the duration of disease in the different heatmap clusters revealed that the blue cluster with the most samples from the active GPA group (71%) had a statistically significant (90% confident interval, *P* = 0.0561) shorter disease duration (median time of 58.5 versus 132 months) compared with the orange cluster. The blue and the orange clusters were the clusters with the most diverse microbiome, whereby the orange cluster is dominated by *Staphylococcus epidermidis*. Column statistics (mean, minimum, maximum, SD) for the blue cluster were 65.71, 5–168, SD 48.16, for the orange cluster were 145.1, 12–552, SD 131, and for the green cluster were 115.8, 5–276, SD 97.4. No statistical differences were identified between disease duration and *Staphylococcus* shotgun-sequenced species (Additional file 5: Figure S5).

## Discussion

We undertook a study to investigate the nasal microbiota in patients with GPA. Bacterial 16S analysis revealed distinctive heatmap clusters. None of the bacterial 16S species were associated with any of the patient groups. This is in contrast to a recent study which reported a lower abundance of *Propionibacterium acnes* and *S. epidermidis* in patients with GPA, but concurred that there were no differences in *S. aureus* abundance among the different groups [13]. The groups had a small number of total participants which might have contributed to non-significant associations. Between-group comparisons using beta diversity analysis revealed that the GPA patients grouped together were different to the healthy controls (HC), suggesting the microbiota of GPA patients undergoes dysbiosis.

Shotgun metagenomic analysis of *Staphylococcus* species offered a deeper insight into the nasal microbiota. *S. aureus* was more abundant in aGPA patients compared with DC or HC, while *S. epidermidis* showed an positive association with HC. PERMANOVA test confirmed differences between aGPA versus HC (*P* = 0.0007) and DC (*P* = 0.0023). Moreover, Spearman’s correlation coefficient showed an association between *S. aureus* and active disease and inactive disease, while *S. epidermidis* was associated with HC. Our metagenomic analysis aligned with our culture data, which also revealed a higher prevalence of *S. aureus* in patients with aGPA (8/12, 66.7%) compared with patients with inGPA (15/44, 34.1%). The lower abundance of *S. epidermidis* in patients with GPA was also recently reported by Rhee et al., in the first study examining the nasal microbiota in GPA [13]. However, unlike in our study and in contrast to one published study [14], they found no association with higher abundance of *S. aureus* in GPA patients. This discrepancy might be explained by the fact that the majority (~ 75%) of GPA cases in the study by Rhee et al. were inactive cases. These results suggest that during disease activity, a dysbiosis of the nasal microbiota is present in patients with GPA.

When we investigated the influence of disease duration on the microbiome, we identified some differences in the 16S sequence data between the blue heatmap cluster, which contained the majority of aGPA patients (71%) with a shorter median time since diagnosis of GPA compared with the orange heatmap cluster which contained only 29% of aGPA patients (29%). All patients in the blue heatmap cluster with active disease received immunosuppression (3 rituximab within the last 6 months, 4 steroids and 1 azathioprine), while one of the active cases in the orange cluster received steroid monotherapy and the other one had no immunosuppressive measure. Both clusters showed a diverse microbiome with a trend for a greater abundance for the *Staphylococcus epidermidis* and *Staphylococcus pseudintermedius* in the orange cluster compared with the blue cluster. The statistical test was significant at 90% confidence level and the sample numbers were low; thus, larger patient groups should be investigated to confirm whether disease duration, disease activity and the prescribed immunosuppression may influence the nasal microbiome in GPA patients.

Diverse mechanisms are implicated in the onset of GPA. *S. aureus* colonisation has emerged as an independent risk factor for disease relapse and higher endonasal activity [6, 7]. Most studies have reported a rate of *S. aureus* nasal colonisation which by far exceeds frequencies observed in the general population [15]. The mechanism leading to higher colonisation rates are so far obscure. Low levels of antibodies against *S. aureus* antigens were found in patients with GPA [9]. These findings were related to surface proteins, secreted proteins and superantigens and superantigen-like proteins, irrespective of the disease state and immunosuppressive treatment [9].

Our results further indicate that *S. aureus* and *S. epidermidis* seem to have an antagonistic relationship. A similar antagonistic relationship has been reported in healthy Danish twins [16], though other studies report no effect [17]. A mechanistic basis for *S. epidermidis* inhibiting *S. aureus* is understood; a subset of *S. epidermidis* strains produce a serine protease, Esp, which inhibits biofilm formation and nasal colonisation by *S. aureus* [18]. Esp is capable of degrading essential proteins implicated in adhesion, biofilm formation, immune and complement evasion, nasal colonisation and human receptor proteins of *S. aureus* including fibronectin and fibrinogen [19]. The relationship between *S. aureus* and *S. epidermidis* in patients with GPA warrants further investigations to decipher factors related to this observed antagonism.

*S. aureus* genomic analysis revealed that no clonal lineage dominated in GPA patients, and there was no evidence of transmission between patients, except in case of a single GPA patient and a HHC. This is similar to previous reports using lower-resolution methods [9]. We also demonstrated in the subset of patients that were repeated sampled, whole genome sequencing showed that *S. aureus* was carried persistently, and the carriers were carrying the same *S. aureus* strain over time, independent of active or inactive disease state. Antibiotic resistance of *S. aureus* isolates was measured in a recent study from the Netherlands [9]. While resistance to penicillin remained stable over time (72.7%) and was comparable with the general population, isolates were more resistant to TMP-SMX (41.4%) and ciprofloxacin (26.7%) over time. This is likely due to the selective pressure of the use of TMP-SMX in GPA patients during the last years [9]. While resistance to penicillin was similar in our cohort, ciprofloxacin and TMP resistance were only found in a single isolate from a single inGPA patient (3.4%) only, indicating that TMP-SMX resistance was rare in our cohort. Only selected cases with GPA in our clinic receive long-term TMP-SMX treatment, which may explain the differences observed in comparison to the Dutch cohort, since long-time treatment is a mainstay of treating localised GPA in their daily practise [9, 20]. During the period of sampling, ten patients with GPA and two with EGPA received TMP-SMX, of whom seven received TMP-SMX as *Pneumocystis jirovecii* prophylaxis, following cyclophosphamide or rituximab treatment.

Among the shotgun-sequenced *Staphylococcus* spp., *Staphylococcus pseudintermedius* was in the top 1% abundance group and accounted for 13% of all *Staphylococcus* spp. *S. pseudintermedius* was detected in the majority of samples (77.1%), but in 13 samples, it was detected with a minimum abundance of 1 % (2% to 12.6%). Considering a cutoff of 0.1% abundance, it was detected in 44% inGPA patients, 41% aGPA patients, 75% HHC, 36% HC and in 46% DC. The presence of *S. pseudintermedius* at first sampling and in the following samples in some patients points towards a persistence in carriage. *S. pseudintermedius* is a commensal and opportunistic pathogen of dogs and cats frequently causing soft tissue and skin infections [21, 22] and is increasingly recognised as zoonosis in humans [23]. Most human infections caused by *S. pseudintermedius* are observed in dog owners, and most frequently skin and soft tissue infections were reported [22]. A recent report from Spain reported dog-to-human transmission in two patients with identical pulsed-field gel electrophoresis patterns, STs, and antimicrobial resistance phenotypes and genotypes [24]. Shotgun metagenomic analysis and 16S PCR highlighted the presence of *S. pseudintermedius* in all groups with no significant differences among the groups. Moreover, the presence of *S. pseudintermedius* could be demonstrated by culture in one patient with sequential samples and in a second patient with single nasal swab; whole genome sequencing of these revealed that in the patient with a sequential swabs, this was indeed a case of persistent carriage [25]. To the best of our knowledge, this is the first study investigating the nasal microbiome which emphasises the presence of *S. pseudintermedius* in human nostrils. It is unclear to date whether or not *S. pseudintermedius* plays a role in GPA disease pathogenesis. We found that in most cases with *S. pseudintermedius* present, the antagonistic relationship between *S. aureus* and *S. epidermidis* was broken, with both species present at lower levels, suggesting *S. pseudintermedius* occupies the same niche.

Visual inspection of the isolate phylogenetic tree with either the nasal bacterial 16S profile or shotgun-sequenced *Staphylococcus* profile did not show a clear association between tree structure and nasal microbiota. Bacterial profiles were not available for all sequenced isolates; thus, larger cohorts are warranted to elucidate whether the nasal microbiota influences the phylogeny of colonising *S. aureus* strains.

Functional analysis of the shotgun sequences revealed 319 functional SEED classifications with a minimum abundance of 0.1% across all samples, and ten of these showed differences among the patients groups. Among the ten identified significant functional SEED annotation pathways, no statistically significant differences were observed between patients with aGPA and inGPA. Thus, the aGPA and inGPA patients were combined for new analysis. Three different SEED annotation pathways were found to be significantly enriched in subjects with GPA compared with the DC (the percentage of coenzyme B12 biosynthesis, histidine degradation and COG0523 histidine genes). A further seven SEED annotation pathways were found to be significantly enriched in GPA patients compared with the HC (the percentage of chorismate synthesis, chorismate biosynthesis in plants, common pathway for synthesis of aromatic compound DAHP synthase to chorismate, coenzyme B12 biosynthesis, B12 cobalamin HMP, methanopterin aromatic and respiratory complex I). Among the significant functional pathways, there was a link between patients with GPA and HHC, further confirming a shared microbiota of individuals living in the same household [26].

Pathway analysis revealed three associations implicated in chorismate synthesis, which is a key intermediate in the synthesis of tryptophan, phenylalanine and tyrosine [27]. Tryptophan depletion and elevation of metabolites are associated with T cell hyporesponsiveness [28, 29]. In myeloperoxidase (MPO)-ANCA vasculitis patients, lower levels of tryptophan were reported compared with the HC [29]. Patients with active vasculitis had the lowest levels, while those with MPO-ANCA vasculitis in remission still had lower levels in comparison to the HC group [29]. Currently, no data is available regarding an association between GPA and tryptophan metabolism. In our analysis, both GPA and DC showed enrichment of genes associated with chorismate synthesis compared with the HC group, which may point towards the importance of chorismate to generate tryptophan.

We also found an association with enrichment of genes involved in the synthesis of vitamin B_12_ in GPA and their HHC compared with the DC and HC. Why there is an enrichment of genes involved in vitamin B_12_ biosynthesis in GPA patients is not clear. Within the human gut microbiota, ~ 80% of bacteria have a requirement for vitamin B_12_, but only 20% can produce it [30], suggesting that there is significant competition for vitamin B_12_, which is likely mirrored in the nose. Indeed, certain gut bacteria have mechanisms for the capture of vitamin B_12_ from host proteins [31]. Therefore, within the nose of GPA patients, vitamin B_12_ might be in limited supply, due to the loss of a bacterial producer that is normally present in the healthy nose, or by changes to the epithelial environment caused by GPA pathogenesis.

Our findings suggest that differences in the nasal metabolic landscape of GPA patients indicate that distinct metabolic niches become available, which can be occupied by normally less abundant species, causing the shift of the taxonomic profile of the GPA patients compared with HC. Evidence for these changes are present in our heatmap analysis. Within the GPA clusters 3, 4, and 5, there are distinct bacterial profiles. There were also differences in the abundances of genes in most of the SEED annotation pathways between clusters 3, 4, and 5. In particular, the abundance of genes in the SEED annotation pathway in cluster 3 is lower than clusters 4 and 5. Cluster 4 is characterised by an increased abundance of *Corynebacterium* species, a lack of *S. aureus* and the greatest abundance of genes in the SEED annotation pathways. These findings, however, need to be interpreted with caution and larger studies are warranted to confirm these results.

Taken together, we observed a higher abundance of *S. aureus* during active disease, while *S. epidermidis* was the dominant *Staphylococcus* spp. in HC. Moreover, we reported for the first time a high abundance of *S. pseudintermedius* in patients and controls which warrants further investigation. The rate of antimicrobial resistances in our *S. aureus* isolates was lower than in previous studies. In general, the impact of changes in the nasal microbiota and outcomes (i.e. relapse rates) needs to be addressed in future longitudinal studies.

## Conclusions

In this study, nasal culture results revealed a higher *S. aureus* positivity in patients with aGPA. Patients with GPA, either active or inactive, grouped together when 16S rRNA profiles were analysed. Performance of shotgun metagenomic analysis highlighted a dominance of *S. aureus* in GPA, while *S. epidermidis* dominated the *Staphylococcus* spp. in HC. SEED functional protein subsystem analysis revealed an association between bacterial dysbiosis and elevated abundance of genes in certain SEED functional groups. We identified *S. pseudintermedius* in a significant proportion of the study population which has not been described in such an abundance in humans before. Further studies investigating the constituents of the nasal microbiota in GPA patients and their metabolic activity in a longitudinal fashion are necessary to draw firm conclusions regarding relapse risk among GPA patients.

## Materials and methods

### Patient cohort

A total of 84 subjects were enrolled for this study. ENT-related disease activity was assessed by a structured clinical investigation [32]. All subjects were Caucasian and were recruited while attending the vasculitis and lupus clinic at Addenbrooke’s Hospital. Clinical characteristics of patients and controls are given in Table 1. Written informed consent was obtained from all patients. This study was conducted in accordance with the ethical principles stated in the Declaration of Helsinki.

### Sample collection, processing, and *Staphylococcus* spp. culture

Nasal swabs (MWE Medical Wire, Sigma Dry Swab Tubed, Corsham, UK) were obtained from both nares according to a pre-defined protocol [33]. A detailed description of swab processing is given in Additional file 11. Antimicrobial susceptibility testing of *S. aureus* was performed on a Vitek 2 instrument (card: AST-P634, bioMérieux, Nürtingen, Germany).

### DNA extraction and whole genome amplification

DNA was extracted from nasal swab fluid using the QIAamp DNA Microbiome Kit. For shotgun sequencing, REPLI-g Mini kit was used for highly uniform whole genome amplification (QIAGEN, Hilden, Germany). A detailed description of the protocol is given in Additional file 11.

### Bacterial 16S rRNA gene library preparation and Illumina MiSeq sequencing

Total DNA was used to perform bacterial 16S PCR reactions using the New England Biolab (NEB) Q5 high-fidelity polymerase kit. We sequenced the bacterial 16S variable V1 V2 gene region with Illumina MiSeq 300 pair-end sequencing technology, which enables 86% full overlap sequencing of the 350 bp V1 V2 gene region from both ends. Further details are given in Additional file 12.

### Shotgun sequencing and cultured isolate sequencing

All 110 samples were shotgun sequenced across seven lanes on the Illumina HiSeq platform using version 4 pair end sequencing. Thirty-two *S. aureus* isolates were sequenced across one lane on the Illumina HiSeq Platform. Libraries for shotgun sequencing and bacterial isolate sequencing were prepared by the Wellcome Sanger Institute core sequencing facility.

### Bioinformatics

#### Bacterial 16S rRNA marker gene analysis

Bacterial 16S rRNA sequences were processed according to the mothur MiSeq SOP. For further details, see the online Additional file 12.

#### Oligotyping and species identification

Oligotyping was used for clustering the high-quality filtered FASTA sequences from the mothur pipeline [34]. The node representative sequence of each oligotype (OTP) was used for species profiling using the ARB analysis - A Software Environment for Sequence Data (version 5.5-org-9167) [35]. We followed a highly stringent in-house pipeline to remove environmental and laboratory contaminants. A detailed description of oligotyping and species identification is given in Additional file 12.

#### Shotgun sequence analysis

The number for raw reads from the shotgun sequencing across the 110 samples was between 7.1 million and 23.5 million reads per samples. High-quality reads were used for contigs and scaffolds generation. Scaffolds were searched using BLASTX search against the NCBI non-redundant nucleotide database and taxonomically annotated. *Staphylococcus* species were used for further analysis in this study. A detailed description of bioinformatics is given in Additional file 12.

#### *Staphylococcus* isolates sequence analysis

Genomic DNA was extracted from *S. aureus* isolates, libraries prepared and 150-bp paired-end sequences determined on an Illumina HiSeq2000 as previously described [36]. Sequence data were assembled using an in-house pipeline [37]. A brief description is given in Additional file 12. The presence of *S. aureus* virulence factors and antibiotic resistance genes were identified using BLAST against the assemblies. For phylogenetic analyses, sequence reads were mapped to a relevant reference genome (ST398 (strain S0385, accession number AM990992) for the overall tree, see Additional file 1: Figure S1) using SMALT (http://www.sanger.ac.uk/science/tools/smalt-0) using the default settings to identify SNPs. For the ST398 phylogeny, the large block of ST8 recombination present in ST398 (S0385 genomic locations: 12252 to 135180) was also removed from the ST398 alignment. SNPs located in mobile genetic elements were removed, and a maximum likelihood tree was created using RAxML using the default settings and 100 bootstrap replicates [38].

### Functional analysis of shotgun metagenomic sequences

For functional analysis of shotgun metagenomic sequence data, we used the functional classification systems of MEGAN using SEED protein subsystem classification. Detailed description are giving in Additional file 12.

### Heatmap analysis

Diamond BLASTX search together with MEGAN analysis of SPAde contigs identified a total of 2891 hits at the species level. Four hundred species with a minimum abundance of 0.01% were used for further analysis. This represent a total of 93.42% MEGAN hits at the species level. Those 400 species were used for heatmap generation together with metadata for the 10 statistically significant SEED functional protein subsystem.

A distance metric was generated with R function “vegist” from the VEGAN package using the “bray” method and Hclust R function from the VEGAN package using the ward. D method was used to cluster the distance matrix. The heatmap was generated with the Heatplus package from R, version 2.26.0.

For easier representation of species on the heatmap, only species with a minimum relative abundance of 2% in at least two samples were used. This cutoff identified 22 of the most abundant species shown on the heatmap. The “cuth” parameter was set to 2.1 which generated five coloured clusters on the hierarchal clustered dendrogram and in the annotation plots. The cuth parameter sets the height at which to cut through the dendrogram to define groups of similar features/samples. We used four sample groups and all ten statistically significant SEED functional protein subsystems for annotation.

### Statistical and visual data analysis

Pattern of beta diversity of bacterial communities, i.e. how the microbiome varies between the different sample groups, was statistically assessed using PERMANOVA. The PERMANOVA test generates a *P* value and a *F* statistic and was performed using the statistical package PAST version 3.09 [39]. Additional information about the PERMANOVA test is given in Additional file 12*.*

Unbiased hierarchical clustering with heatmap generation was generated using the R package “Heatplus” [40]. Stack bar chart presented next to the heatmaps was generated in Apple Keynote version 6.6.2. The proportional abundance of species used for the stack bar charts was calculated in Microsoft Excel for Mac, version 15.41.

The rank-based indirect gradient analysis “NMDS” was used for the visualisation of taxonomic differences (beta diversity differences) between the different groups. Additional information about NMDS is given in Additional file 12. In addition to NMDS, we also used CA, an indirect gradient analysis based on a multivariate statistical technique similar to principal component analysis that provides a means of displaying or summarising a set of data in a two-dimensional graphical form.

Spearman’s rho coefficient analyses were performed with PAST3 [39] to identify patterns of association of bacterial OTP species with particular sample groups. Scatter plot presentation of samples and non-parametric Kruskal-Wallis test with Dunn’s multiple comparison test was done in GraphPad Prism 6 for Mac OS X, version 6.0h.

## Supplementary information

**Additional file 1: Figure S1.** 31 cultured *Staphylococcus* isolates were sequenced and their assembled genomes were used for phylogenetic tree construction. **a**. Bacterial 16S marker gene sequenced species were matched with cultured *S. aureus* isolates in their phylogenetic tree. The top 22 species with a minimum abundance of 0.1% were used for the stack bar chart presentation. The top 22 species represented 98.91% of all reads. Please note: a bacterial 16S profile was not available from all samples from which an isolate was sequenced. **b**. Shotgun sequenced *Staphylococcus* species were matched with cultured *S. aureus* isolates in their phylogenetic tree. The top 28 taxa with a minimum abundance of 0.1% were used for the stack bar chart presentation. The top 28 taxa represented 97.25% of all reads. Please note: A *Staphylococcus* profile was not available from all samples from which an isolate was sequenced. (PDF 8765 kb)

**Additional file 2: Figure S2.** NMDS analysis of bacterial 16S marker gene sequenced species. **a**. NMDS plot reveals that healthy controls (HC) form a cluster separating them from inactive [green ellipse] and active GPA patients [orange ellipse]. The two disease controls (EGPA) are within the inGPA and aGPA clusters. The overall cluster separation is not statistically different (PERMANOVA test: *P* value > 0.05). **b**. The inGPA and aGPA samples were grouped into one cluster [GPA cluster, orange ellipse]. PERMANOVA test between the three groups revealed that samples from the combined GPA patients are statistically different from the healthy control (HC) samples (PERMANOVA *P* value = 0.039, F value = 1.739). (PDF 155 kb)

**Additional file 3: Figure S3.** Taxonomic annotation of longitudinal case studies using bacterial 16S marker gene sequenced species. Bacterial diversity in six patients with follow up sampling one month (*n* = 6) and 3 months later (*n* = 1) is shown. In two case studies, household controls at the initial sampling time point (*n* = 2) and one month later (*n* = 1) were also available. For comparisons, seven healthy controls are shown at the bottom of the figure. The abundance of the top 27 species with a minimum 0.1% contribution in samples from longitudinal case studies with matching species from healthy controls are shown on the x axis. (PDF 629 kb)

**Additional file 4: Figure S4.** Heatmap analysis with annotation for disease duration in months and active GPA and inactive GPA disease stage using top 28 species with a minimum abundance of 0.5% in at least one sample in the bacterial 16S rRNA dataset. (PDF 75 kb)

**Additional file 5: Figure S5.** Heatmap analysis with annotation for disease duration in months and active GPA, inactive GPA, and disease control using top 18 *Staphylococcus* species with a minimum abundance of 0.5% in at least one sample in the shotgun sequenced dataset. (PDF 83 kb)

**Additional file 6: Table S1**. Antimicrobial susceptibility testing of *S. aureus* isolates. (DOCX 18 kb)

**Additional file 7: Table S2**. *S. aureus* isolates and relevant metadata. (XLSX 13 kb)

**Additional file 8: Table S3**. Relative abundance of *S. aureus* in the bacterial 16S dataset and in the shot gun metagenomic dataset. (DOCX 16 kb)

**Additional file 9: Table S4.** Positive MEGAN hits for shot gun sequenced Staphylococcus species. (XLSX 10 kb)

**Additional file 10: Table S5.** 319 SEED functional protein subsystem with minimum abundance of 0.01% in base line samples. (XLSX 181 kb)

**Additional file 11.** Supplementary Material and Method (DOCX 92 kb)

**Additional file 12.** Bioinformatics Analysis (DOCX 45 kb)

## Data Availability

European Nucleotide Archive (ENA) study accession: ERP016546.

## Abbreviations

AB: Antibiotic
aGPA: Active GPA
ANCA: Anti-neutrophil cytoplasm antibody
CA: Correspondence analysis
DC: Disease control
DNA: Deoxyribose nucleic acid
EGPA: Eosinophilic granulomatosis with polyangiitis
ENT: Ear, nose and throat
GPA: Granulomatosis with polyangiitis
HC: Healthy control
HHC: Healthy household control
inGPA: Inactive GPA
MALDI-TOF: Matrix-assisted laser desorption/ionisation–time-of-flight mass spectrometry
MLST: Multilocus sequence type
MPO: Myeloperoxidase
MRSA: Methicillin-resistant *Staphylococcus aureus*
NMDS: Non-metric multidimensional scaling
OTP: Oligotype
PCR: Polymerase chain reaction
PERMANOVA: Permutational multivariate analysis of variance
RNA: Ribonucleic acid
SNP: Single-nucleotide polymorphism
ST: Sequence type
TMP-SMX: Trimethoprim-sulfamethoxazole
WGS: Whole genome sequencing
