# Supplementary material for: The composition and functional protein subsystems of the human nasal microbiome in granulomatosis with polyangiitis: a pilot study
Source: Microbiome. 2019 Oct 22;7:137. doi: 10.1186/s40168-019-0753-z (PMC6806544; doi:10.1186/s40168-019-0753-z)
Supplement: Supplementary file 1 — Additional file 1: Figure S1. 31 cultured Staphylococcus isolates were sequenced and their assembled genomes were used for phylogenetic tree construction. a. Bacterial 16S marker gene sequenced species were matched with cultured S. aureus isolates in their phylogenetic tree. The top 22 species with a minimum abundance of 0.1% were used for the stack bar chart presentation. The top 22 species represented 98.91% of all reads. Please note: a bacterial 16S profile was not available from all samples from which an isolate was sequenced. b. Shotgun sequenced Staphylococcus species were matched with cultured S. aureus isolates in their phylogenetic tree. The top 28 taxa with a minimum abundance of 0.1% were used for the stack bar chart presentation. The top 28 taxa represented 97.25% of all reads. Please note: A Staphylococcus profile was not available from all samples from which an isolate was sequenced. (PDF 8765 kb) [file 40168_2019_753_MOESM1_ESM.pdf]

Supplementary Figure 1 a. Bacterial 16S marker gene sequenced species were matched with cultured *Staphylococcus aureus* isolates in their phylogenetic tree

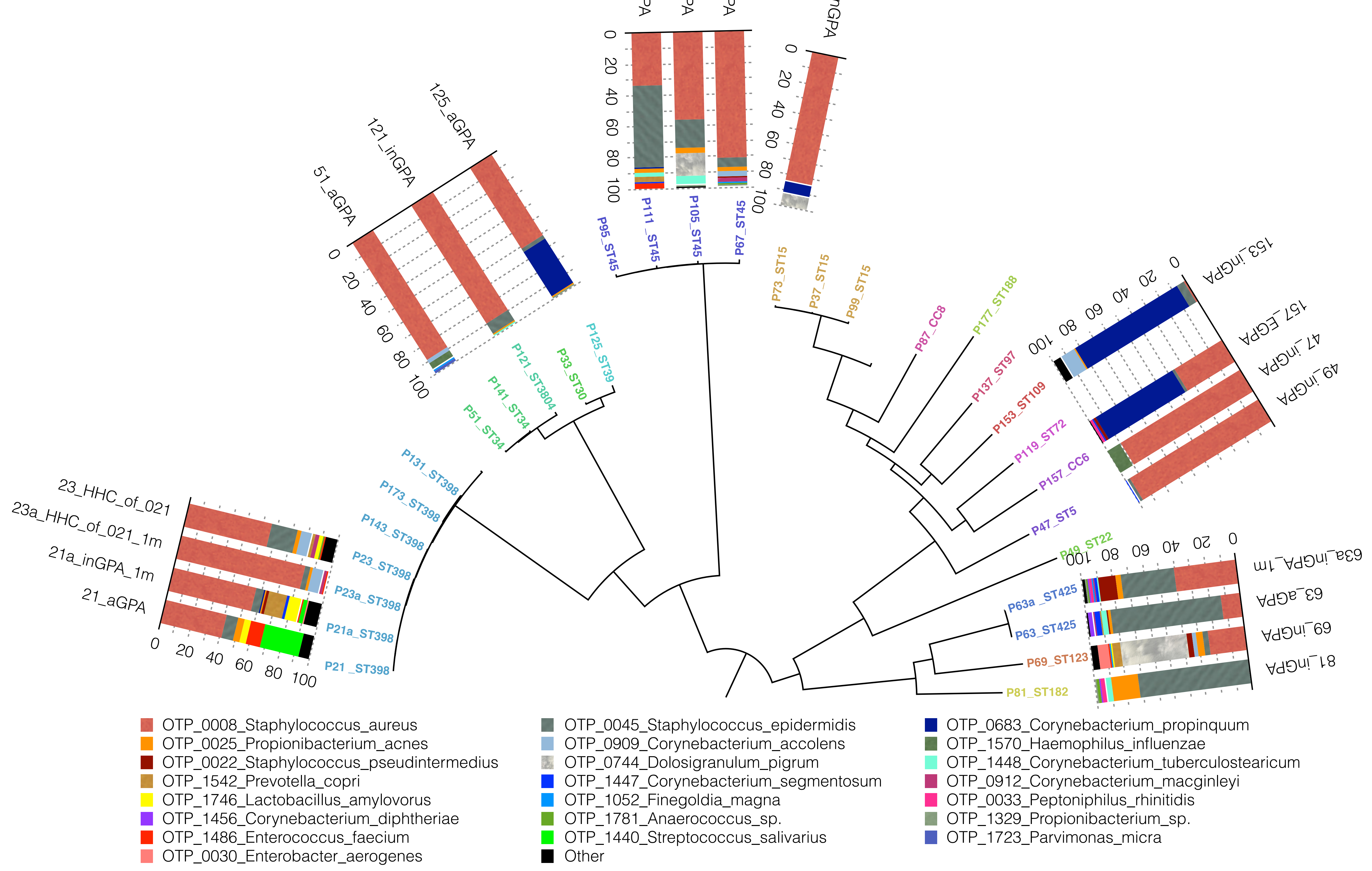

Supplementary Figure 1 b. Shotgun sequenced *Staphylococcus* species were matched with cultured *Staphylococcus aureus* isolates in their phylogenetic tree

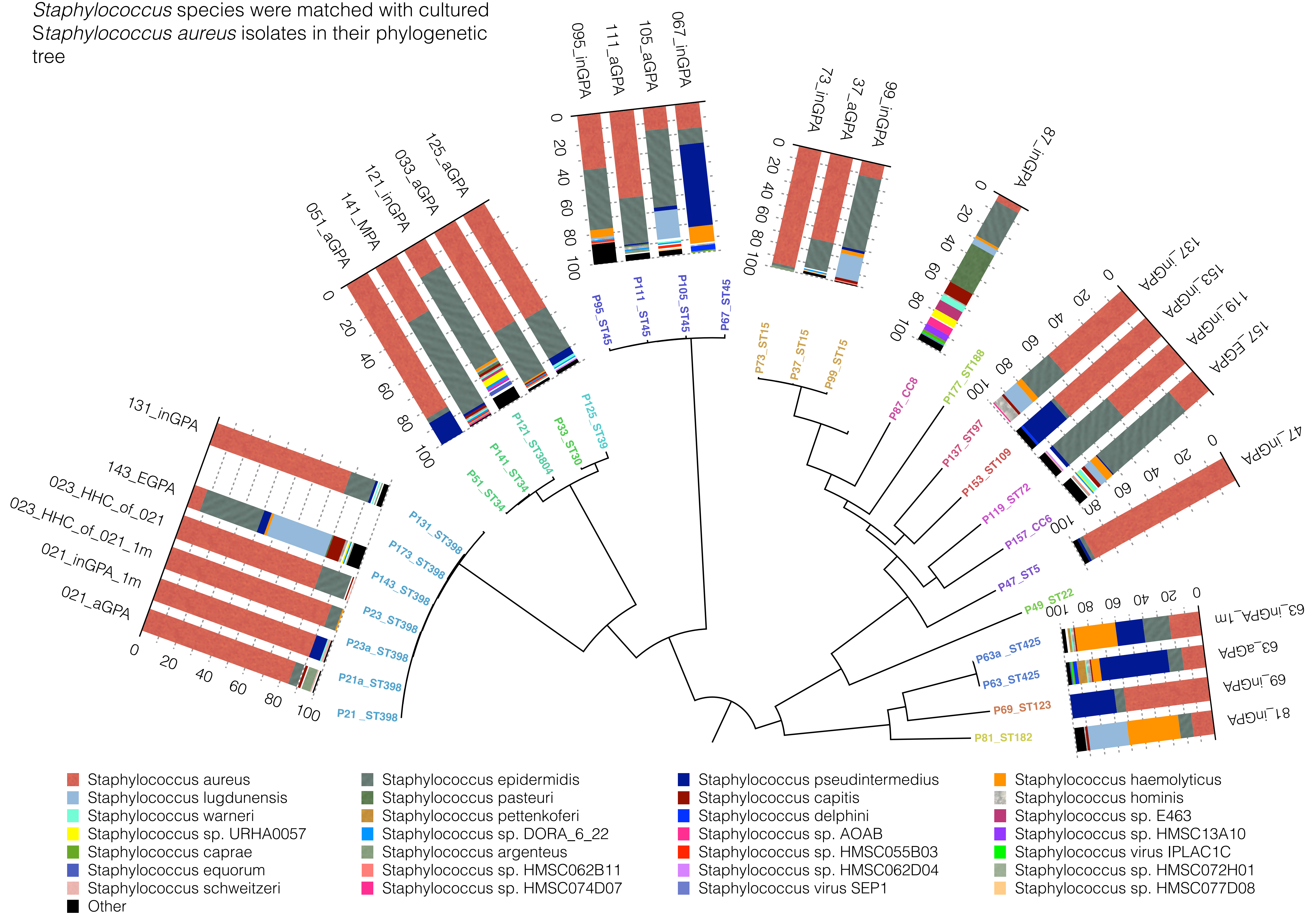

Supplementary Figure 1
31 cultured *Staphylococcus* isolates were sequenced and their assembled genomes were used for phylogenetic tree construction.

Supplementary Figure 1 a
Bacterial 16S marker gene sequenced species were matched with cultured *Staphylococcus* aureus isolates in their phylogenetic tree. The top 22 species with a minimum abundance of 0.1% were used for the stack bar chart presentation. The top 22 species represented 98.91% of all reads. Please note: a bacterial 16S profile was not available from all samples from which an isolate was sequenced.

Supplementary Figure 1 b
Shotgun sequenced *Staphylococcus* species were matched with cultured S*taphylococcus aureus* isolates in their phylogenetic tree
The top 28 taxa with a minimum abundance of 0.1% were used for the stack bar chart presentation. The top 28 taxa represented 97.25% of all reads. Please note: a *Staphylococcus* profile was not available from all samples from which an isolate was sequenced.
