# Supplementary material for: The composition and functional protein subsystems of the human nasal microbiome in granulomatosis with polyangiitis: a pilot study
Source: Microbiome. 2019 Oct 22;7:137. doi: 10.1186/s40168-019-0753-z (PMC6806544; doi:10.1186/s40168-019-0753-z)
Supplement: Supplementary file 2 — Additional file 2: Figure S2. NMDS analysis of bacterial 16S marker gene sequenced species. a. NMDS plot reveals that healthy controls (HC) form a cluster separating them from inactive [green ellipse] and active GPA patients [orange ellipse]. The two disease controls (EGPA) are within the inGPA and aGPA clusters. The overall cluster separation is not statistically different (PERMANOVA test: P value > 0.05). b. The inGPA and aGPA samples were grouped into one cluster [GPA cluster, orange ellipse]. PERMANOVA test between the three groups revealed that samples from the combined GPA patients are statistically different from the healthy control (HC) samples (PERMANOVA P value = 0.039, F value = 1.739). (PDF 155 kb) [file 40168_2019_753_MOESM2_ESM.pdf]

Supplementary Figure 2. NMDS analysis of bacterial 16S marker gene sequenced species

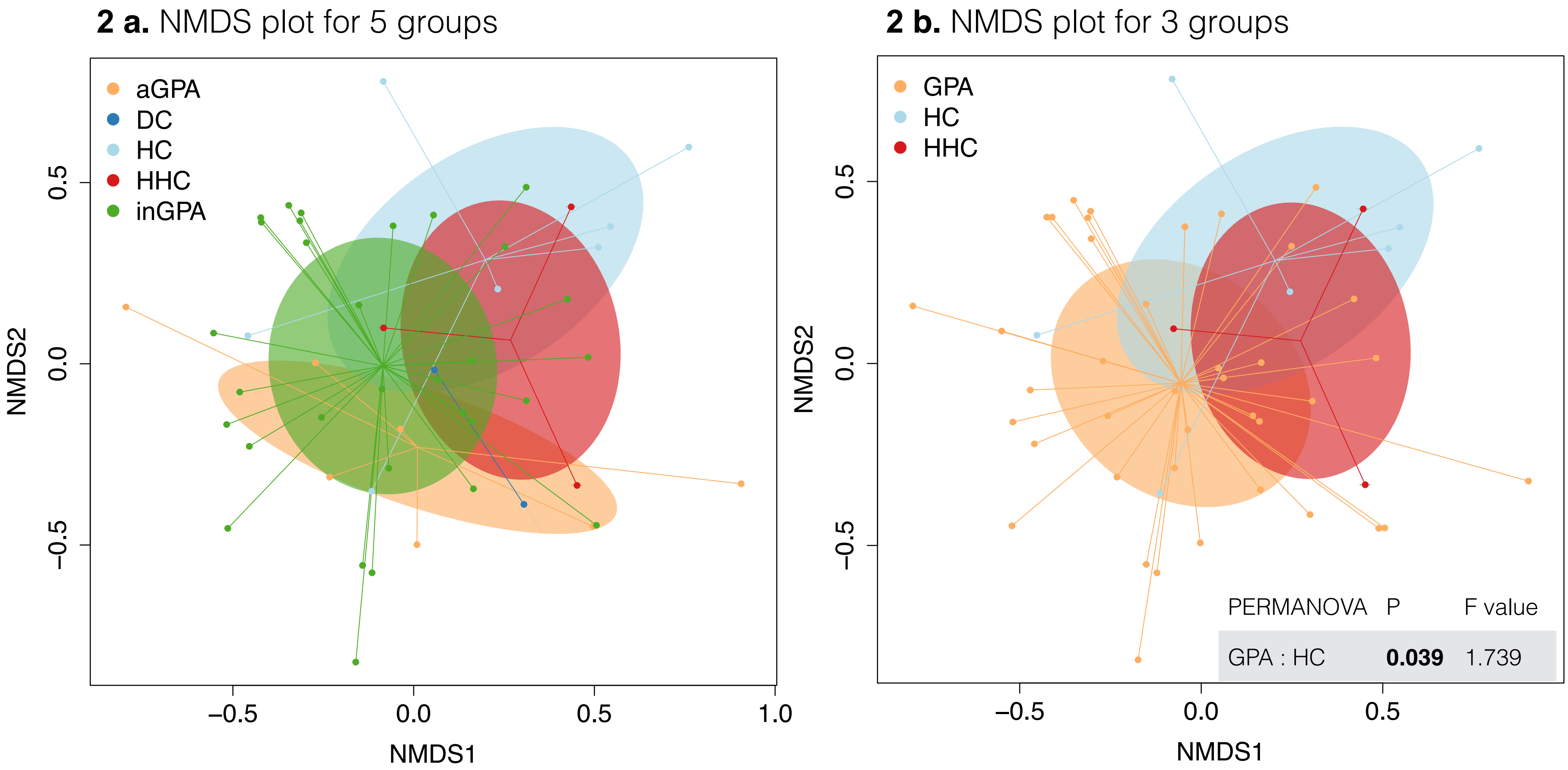

Supplementary Figure 2 a.
NMDS plot reveals that healthy controls (HC) form a cluster most separated from the inactive GPA patients [green ellipse] and active GPA patients [orange ellipse]. The two disease controls (DC) samples are within the inGPA and aGPA clusters. The overall cluster separation is not statistically different (PERMANOVA test: P value > 0.05).

Supplementary figure 2 b.
The inGPA samples and aGPA samples were grouped into one cluster [GPA cluster, orange ellipse]. PERMANOVA test between the three groups revealed that samples from the combined GPA patients are statistically different from the healthy control (HC) samples (PERMANOVA P value = 0.039, F value = 1.739).
