# Supplementary material for: The composition and functional protein subsystems of the human nasal microbiome in granulomatosis with polyangiitis: a pilot study
Source: Microbiome. 2019 Oct 22;7:137. doi: 10.1186/s40168-019-0753-z (PMC6806544; doi:10.1186/s40168-019-0753-z)
Supplement: Supplementary file 3 — Additional file 3: Figure S3. Taxonomic annotation of longitudinal case studies using bacterial 16S marker gene sequenced species. Bacterial diversity in six patients with follow up sampling one month (n = 6) and 3 months later (n = 1) is shown. In two case studies, household controls at the initial sampling time point (n = 2) and one month later (n = 1) were also available. For comparisons, seven healthy controls are shown at the bottom of the figure. The abundance of the top 27 species with a minimum 0.1% contribution in samples from longitudinal case studies with matching species from healthy controls are shown on the x axis. (PDF 629 kb) [file 40168_2019_753_MOESM3_ESM.pdf]

Supplementary Figure 3. Taxonomic annotation of longitudinal case studies using bacterial 16S marker gene sequenced species

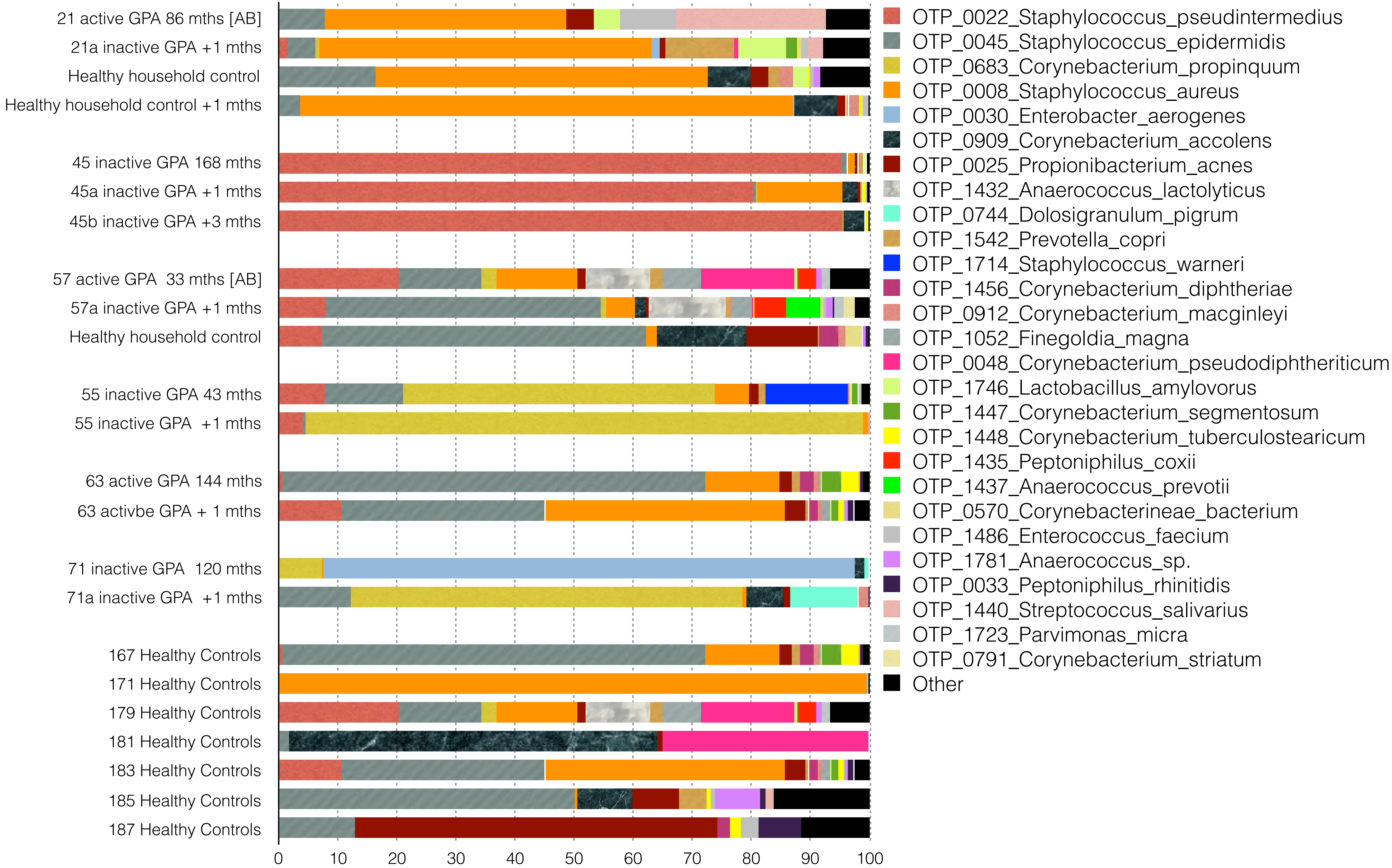

Proportional abundance of OTP species with minimum 0.1% contribution in samples from longitudinal case studies with matching species from healthy controls

Supplementary Figure 3. Taxonomic annotation of longitudinal case studies using bacterial 16S marker gene sequenced species

Bacterial diversity in six patients with follow up sampling one month (n = 6) and 3 months later (n = 1) is shown. In two case studies, household controls at initial sampling time point (n = 2) and one months later (n = 1) were also available. For comparisons, seven healthy controls are shown at the bottom of the figure. The abundance of the top 27 species with a minimum 0.1% contribution in samples from longitudinal case studies with matching species from healthy controls are shown on the x axis.
