# Supplementary material for: The composition and functional protein subsystems of the human nasal microbiome in granulomatosis with polyangiitis: a pilot study
Source: Microbiome. 2019 Oct 22;7:137. doi: 10.1186/s40168-019-0753-z (PMC6806544; doi:10.1186/s40168-019-0753-z)

Supplementary Figure 4. Heatmap analysis with annotation for disease duration in months and active GPA and inactive GPA disease stage using top 28 species with a minimum abundance of 0.5% in at least one sample in the **bacterial 16S rRNA dataset.**

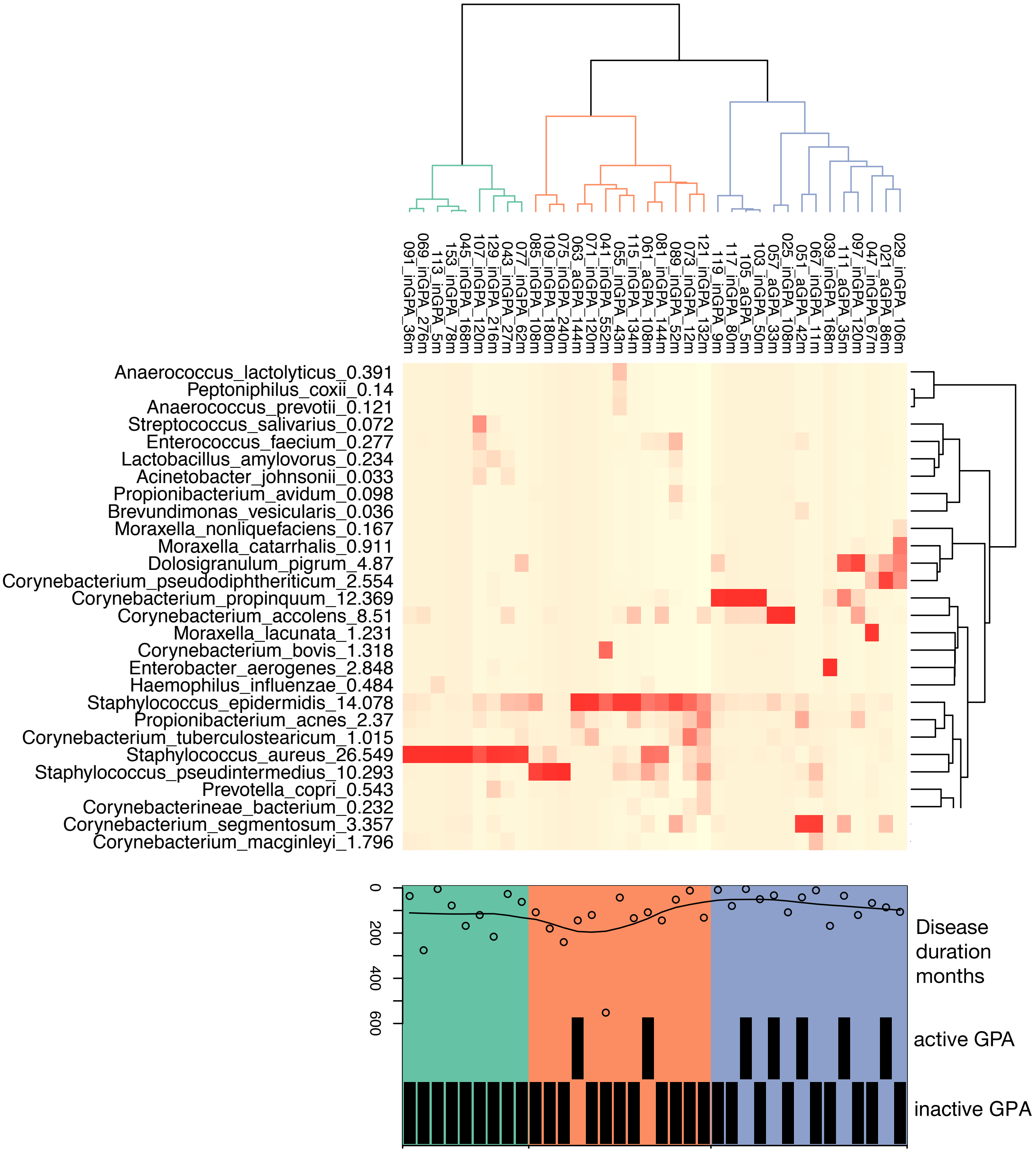

Supplement: Supplementary file 4 — Additional file 4: Figure S4. Heatmap analysis with annotation for disease duration in months and active GPA and inactive GPA disease stage using top 28 species with a minimum abundance of 0.5% in at least one sample in the bacterial 16S rRNA dataset. (PDF 75 kb) [file 40168_2019_753_MOESM4_ESM.pdf]
