# Supplementary material for: The composition and functional protein subsystems of the human nasal microbiome in granulomatosis with polyangiitis: a pilot study
Source: Microbiome. 2019 Oct 22;7:137. doi: 10.1186/s40168-019-0753-z (PMC6806544; doi:10.1186/s40168-019-0753-z)

Supplementary Figure 5. Heatmap analysis with annotation for disease duration in months and active GPA, inactive GPA, and disease control using top 18 *Staphylococcus* species with a minimum abundance of 0.5% in at least one sample in the shotgun sequenced dataset.

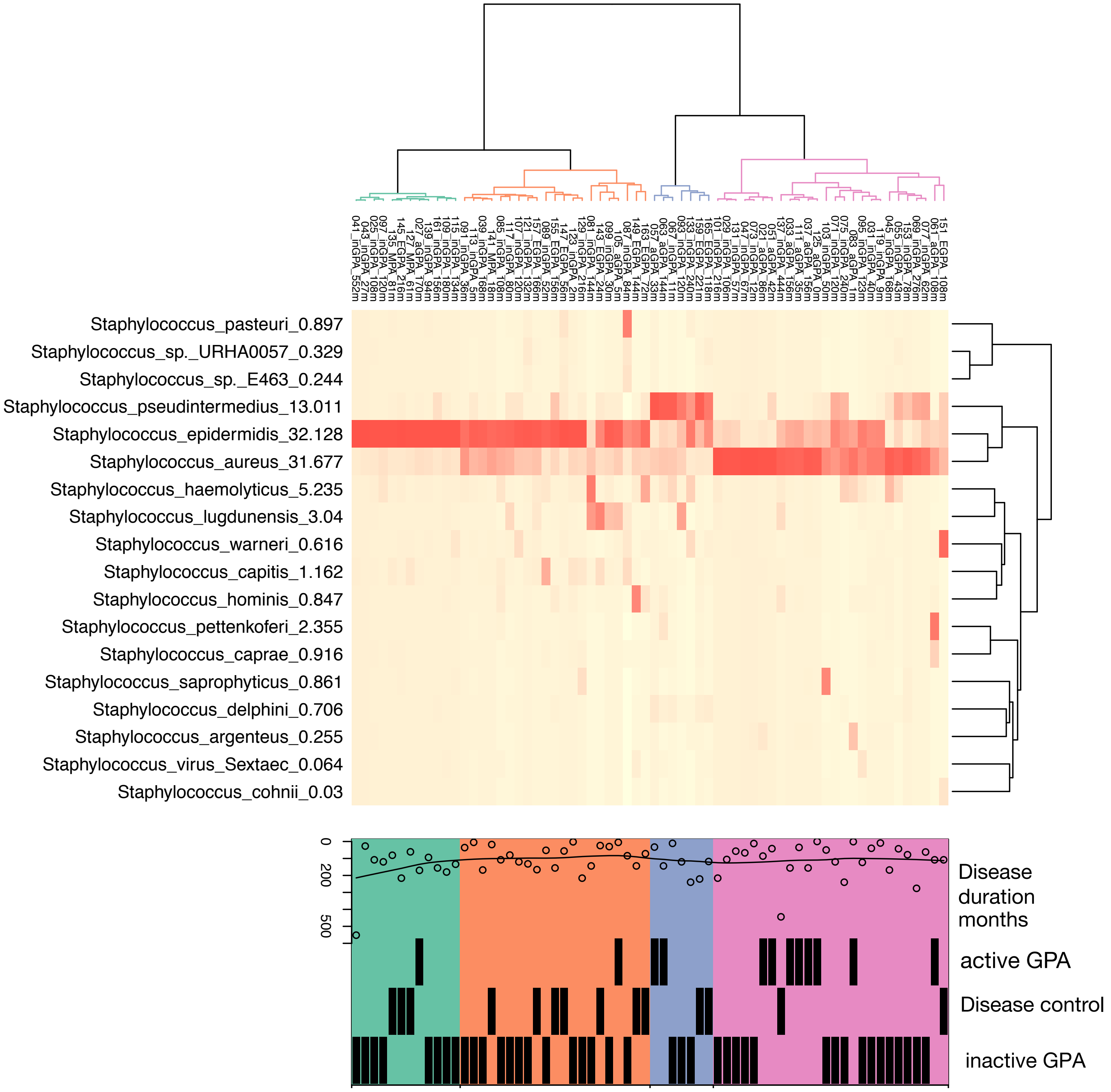

Supplement: Supplementary file 5 — Additional file 5: Figure S5. Heatmap analysis with annotation for disease duration in months and active GPA, inactive GPA, and disease control using top 18 Staphylococcus species with a minimum abundance of 0.5% in at least one sample in the shotgun sequenced dataset. (PDF 83 kb) [file 40168_2019_753_MOESM5_ESM.pdf]
