# Supplementary material for: The composition and functional protein subsystems of the human nasal microbiome in granulomatosis with polyangiitis: a pilot study
Source: Microbiome. 2019 Oct 22;7:137. doi: 10.1186/s40168-019-0753-z (PMC6806544; doi:10.1186/s40168-019-0753-z)
Supplement: Supplementary file 6 — Additional file 6: Table S1. Antimicrobial susceptibility testing of S. aureus isolates. (DOCX 18 kb) [file 40168_2019_753_MOESM6_ESM.docx]

**Supplementary Table 2**: Antimicrobial susceptibilty testing of S. aureus isolates

|  | Benzylpenicillin | Oxacillin | Genta | Cipro | Erythromycin | Clindamycin | Linezolid | Daptomycin | Teicoplanin | Vanco | Tetracycline | Nitrofurantoin | FA | Mupi | CMP | RMP | TMP |
| --- | --- | --- | --- | --- | --- | --- | --- | --- | --- | --- | --- | --- | --- | --- | --- | --- | --- |
| 0021 | + | - | - | - | + | - | - | - | - | - | - | - | - | - | - | - | - |
| 0021-1 | + | - | - | - | + | - | - | - | - | - | - | - | - | - | - | - | - |
| 0023 | + | - | - | - | + | - | - | - | - | - | - | - | - | - | - | - | - |
| 0023-1 | + | - | - | - | + | - | - | - | - | - | - | - | - | - | - | - | - |
| 0029 | - | - | - | - | - | - | - | - | - | - | - | - | - | - | - | - | - |
| 0033 | + | - | - | - | - | - | - | - | - | - | - | - | - | + | - | - | - |
| 0037 | + | - | - | - | - | - | - | - | - | - | - | - | - | - | - | - | - |
| 0047 | - | - | - | - | - | - | - | - | - | - | - | - | + | - | - | - | - |
| 0049 | + | - | - | + | - | - | - | - | - | - | - | - | - | - | - | - | + |
| 0051 | + | - | - | - | - | - | - | - | - | - | - | - | - | + | - | - | - |
| 0063 | - | - | - | - | - | - | - | - | - | - | - | - | - | - | - | - | - |
| 0063-1 | - | - | - | - | - | - | - | - | - | - | - | - | - | - | - | - | - |
| 0067 | + | - | - | - | - | - | - | - | - | - | - | - | - | - | - | - | - |
| 0069 | + | - | - | - | - | - | - | - | - | - | - | - | + | - | - | - | - |
| 0073 | + | - | - | - | - | - | - | - | - | - | - | - | - | - | - | - | - |
| 0081 | + | - | - | - | - | - | - | - | - | - | - | - | - | - | - | - | - |
| 0087 | - | - | - | - | - | - | - | - | - | - | - | - | - | - | - | - | - |
| 0095 | - | - | - | - | - | - | - | - | - | - | - | - | - | + | - | - | - |
| 0099 | - | - | - | - | - | - | - | - | - | - | - | - | - | - | - | - | - |
| 0105 | + | - | - | - | - | - | - | - | - | - | - | - | - | - | - | - | - |
| 0111 | + | - | - | - | - | - | - | - | - | - | - | - | - | - | - | - | - |
| 0119 | + | - | - | - | + | - | - | - | - | - | + | - | - | - | - | - | - |
| 0121 | + | - | - | - | - | - | - | - | - | - | - | - | - | - | - | - | - |
| 0125 | + | - | - | - | - | - | - | - | - | - | - | - | - | - | - | - | - |
| 0131 | + | - | - | - | - | - | - | - | - | - | - | - | - | - | - | - | - |
| 0137 | + | - | - | - | - | - | - | - | - | - | - | - | - | + | - | - | - |
| 0141 | + | - | - | - | - | - | - | - | - | - | - | - | - | - | - | - | - |
| 0143 | + | - | - | - | + | - | - | - | - | - | - | - | - | - | - | - | - |
| 0153 | + | - | - | - | + | - | - | - | - | - | - | - | - | - | - | - | - |
| 0157 | - | - | - | - | + | - | - | - | - | - | - | - | - | - | - | - | - |
| 0173 | + | - | - | - | + | - | - | - | - | - | - | - | - | - | - | - | - |
| 0177 | + | - | - | - | - | - | - | - | - | - | + | - | - | - | - | - | - |
