# Supplementary material for: The composition and functional protein subsystems of the human nasal microbiome in granulomatosis with polyangiitis: a pilot study
Source: Microbiome. 2019 Oct 22;7:137. doi: 10.1186/s40168-019-0753-z (PMC6806544; doi:10.1186/s40168-019-0753-z)
Supplement: Supplementary file 8 — Additional file 8: Table S3. Relative abundance of S. aureus in the bacterial 16S dataset and in the shot gun metagenomic dataset. (DOCX 16 kb) [file 40168_2019_753_MOESM8_ESM.docx]

| **Patient** | **Relative *S. aureus* abundance (16S)** | **Relative S. aureus abundance (shot gun)** | **Relapse** | **Time to relapse (months)** | **Follow-up period (months)** | **Off-treatment (sampling)** |
| --- | --- | --- | --- | --- | --- | --- |
| **0025** | 1,848198526 | 5,735294118 | no |  | 36 | yes |
| **0029** | 0,266193434 | 79,70731707 | no |  | 36 | yes |
| **0039** | 0,264388855 | 28,38487973 | no |  | 36 | no |
| **0041** | 1,666854375 | 2,494331066 | no |  | 6 | yes |
| **0043** | 56,23529412 | 4,849279161 | yes | 8 | 14 (death) | no |
| **0045** | 95,4028436 | 59,77496484 | yes | 30 | 36 | no |
| **0047** | 0,072979383 | 92,74546592 | no |  | 36 | no |
| **0049** | 0,295420975 | 5,79297246 | yes | 32 | 36 | no |
| **0055** | 4,913928013 | 45,58063639 | no |  | 36 | no |
| **0065** | 83,77000425 | 23,14949202 | yes | 28 | 36 | no |
| **0067** | 3,759743237 | 16,60047657 | yes | 9 | 36 | no |
| **0069** | 83,3315795 | 61,03542234 |  |  | 0 | no |
| **0071** | 0,525578136 | 34,09090909 | no |  | 36 | no |
| **0073** | 6,30174793 | 96,01449275 | yes | 27 | 36 | no |
| **0075** | 1,130073801 | 34,45945946 | yes | 24 | 36 | yes |
| **0077** | 56,39097744 | 52,27272727 | no |  | 36 | no |
| **0081** | 29,60784314 | 15,33840609 | no |  | 36 | yes |
| **0085** | 2,965958881 | 24,85981308 | yes | 5 | 36 | no |
| **0089** | 0,742942051 | 5,282112845 | yes | 1 | 36 | yes |
| **0091** | 80,77275971 | 39,43661972 | yes | 14 | 36 | no |
| **0097** | 0,031123561 | 9,090909091 | no |  | 36 | no |
| **0103** | 1,588447653 | 33,52626892 | no |  | 36 | yes |
| **0107** | 40,7960199 | 19,56521739 | yes | 15 | 36 | no |
| **0109** | 14,31409337 | 8,370702541 | yes | 11 | 36 | no |
| **0113** | 90,23778469 | 23,75 | no |  | 4 | no |
| **0115** | 1,736227045 | 6 | yes | 31 | 36 | no |
| **0117** | 1,19047619 | 23,02158273 | yes | 20 | 36 | no |
| **0119** | 2,159827214 | 47,10300429 | no |  | 36 | no |
| **0121** | 9,437751004 | 16,90821256 | yes | 10 | 36 | yes |
| **0129** | 56,23673662 | 10,23622047 | no |  | 36 | yes |
| **0153** | 96,48876404 | 70,90909091 | no | 1 | 36 | yes |

**Supplementary Table 4**: Relative abundance of S. aureus in the bacterial 16S dataset and in the shot gun metagenomic dataset

**Supplementary Table 4.** Patient numbers (consecutive), the relative abundance of *Staphylococcus aureus* (as measured by 16S analysis), analysis of *S. aureus* among the retrieved *Staphylococcus* spp. (as measured by shot gun sequencing), the follow-up period including the characteristics (future relapse) and the respective follow-up (censored at 36 months) are given. We highlighted if patients have been off treatment during sample collection.
