# Supplementary material for: The composition and functional protein subsystems of the human nasal microbiome in granulomatosis with polyangiitis: a pilot study
Source: Microbiome. 2019 Oct 22;7:137. doi: 10.1186/s40168-019-0753-z (PMC6806544; doi:10.1186/s40168-019-0753-z)
Supplement: Supplementary file 11 — Additional file 11. Supplementary Material and Method (DOCX 92 kb) [file 40168_2019_753_MOESM11_ESM.docx]

**Supplementary Material and Method**

**2.2 Sample collection, processing, and *Staphylococcus spp.* culture**

Nasal swabs (MWE Medical Wire, Sigma Dry Swab Tubed, Corsham, UK) were obtained from both nares according to a pre-defined protocol ([13](#_ENREF_13)). The cultivation of colonies from swabs and their analysis are outlined in the Supplementary material and method section. Swabs were vortexed and used to inoculate high-salt (7.5%) nutrient broths (All media: Oxoid Broth, Basingstoke, UK) and statically incubated overnight at 37ºC. Broth was then used to inoculate a Brilliance Staph 24 plate and incubated for 24 hours at 37ºC. After 24 hours representative blue single colonies (putative *S. aureus*) were picked and streaked to purity on Columbia blood agar and incubated for 24 hours at 37ºC for further analysis. On any plates that didn’t produce blue colonies, representative white colonies of each morphology present were also picked and streaked to purity on Columbia blood agar. Representative colonies from each Columbia plate were then analysed by matrix assisted laser desorption ionization-time of flight mass spectrometry ([14](#_ENREF_14)).

**2.3 DNA extraction and whole genome amplification**

DNA extraction was performed using QIAamp UCP Pathogen Mini Kit according to the manufacturer instruction with additional/replacement steps highlighted in grey. (QIAGEN Hilden, Germany).

Procedure for 1 ml sample (DNA extraction)

1. Add 500 µl Buffer AHL to 1 ml of sample in a 2ml tube* and incubate for 30 minutes at room temperature with end-over-end rotation.
2. Centrifuge (10,000 x g for 10 minutes) and remove supernatant
3. Add 190 µl Buffer RDD and 2.5 µl Benzonase, mix well and incubate (37° for 30 minutes at 600 rpm in a heating block or a water bath)
4. Add 20 µl Proteinase K and incubate at 56° for 30 minutes at 600 rpm in a heating block or a water bath
5. Briefly spin tube to remove condensation, addition of 200 µl Buffer ATL (containing reagent DX). Mix well and transfer in Pathogen ~~Lysis Tube L~~

Please note: instead of Pathogen Lysis Tube L, we used Pathogen Lysis Tube S from Qiagen, which should give higher yield and was supplied extra by QIAgen.

1. Lysis of bacterial cells: Place the Pathogen Lysis Tube S on a vortexer with a microtube foam insert and vortex for 10 minutes at maximum speed (note: avoid heating of samples during lysis)
2. Centrifuge (10,000 x g for 1 minute) to reduce the amount of foam (mix carefully and transfer the supernatant to a fresh microcentrifuge tube)
3. Add 40 µl Proteinase K, mix by vortexing, and incubate at 56° for 30 minutes at 600 rpm in a heating block or water bath
4. Add 200 µl Buffer APL2. Mix by pulse vortexing for 30 seconds
5. Incubate at 70° for 10 minutes and briefly spin the tube
6. Add 200 µl ethanol to the lysate. Mix thoroughly by pulse vortexing for 15-30 seconds
7. Carefully apply up to 700 µl of the mixture from step 11 to the QIAamp UCP Mini Column without wetting the rim. Close the cap and centrifuge at 6,000 x g for 1 minute
8. Discard the flow-through. Put the column back into the collection tube to repeat step 12 with any remaining mixture from step 11 (note: flow-through containing Buffer APL2 or Buffer AW1 is not compatible with bleach)
9. Transfer the QIAamp UCP Mini Column to a fresh collection tube. Carefully open the cap and add 500 μl Buffer AW1 without wetting the rim. Close the cap and centrifuge at 6,000 x g for 1 minute. Place the QIAamp UCP Mini Column into a fresh 2 ml collection tube and discard the filtrate (note: comment see above)
10. Carefully open the QIAamp UCP Mini Column and add 500 μl Buffer AW2 without wetting the rim. Centrifuge at full speed (20,000 x g) for 3 minutes
11. Place the QIAamp UCP Mini Column into a fresh 2 ml collection tube. Discard the filtrate. Centrifuge at full speed (20,000 x g) for 1 minute
12. Place the QIAamp UCP Mini Column into a fresh 1.5 ml tube and apply 50 μl Buffer AVE directly onto the center of the membrane. Close the lid and incubate at room temperature for 5 minutes. Repeat addition of 50 μl Buffer AVE step and incubate at room temperature for 5 minutes.
13. Centrifuge at 6,000 x g for 1 minute to elute the DNA

Whole genome amplification (WGA)

WGA was performed using QIAamp REPLI-g Mini kit according to the manufacturer instruction with additional/replacement steps highlighted in grey. (QIAGEN Hilden, Germany).

REPLI-g Mini DNA Polymerase should be thawed on ice. All other components can be thawed at room temperature.

2.5 µl template DNA is used

5 µl template DNA is used (We used the 5 µl template DNA pipeline)

Things to do before starting:

Prepare Buffer DLB by adding 500 μl nuclease-free water to the tube. Mix thoroughly and centrifuge briefly (DLB can be stored for 6 months at –20°C). Vortex reagents before using. We used a modified WGA protocol according to Oyola SO, et al. (1)

All tubes were UV-treated before WGA reactions were performed in 0.2 ml PCR tubes

Step 1 Buffer D1 (denaturation buffer) and Step 2 Buffer N1 (neutralization buffer)

The total volume for 15 and **7 reactions** is given below:

D1 (original protocol):

D1 (modified WGA protocol):

The working solution was prepared by mixing the stock solution (= reconstituted Buffer DLB) and nuclease-free water in the ratio of 1:3.5.

N1 (original protocol):

N1 (modified WGA protocol):

To the above mentioned protocol (1:5.7) addition of 300 mM tetramethylammonium chloride (TMAC)* – improved quality of MDA product (coverage and base composition) These step was not performed.

Step 3 2.5 µl or **5 µl** template into a microcentrifuge

Step 4 Add 2.5 µl or **5 µl Buffer D1**, mix by vortexing and centrifuge briefly.

Step 5 Incubate the samples at room temperature for 3 minutes.

Step 6 Add 5 µl or **10 µl Buffer N1**, mix by vortexing and centrifuge briefly.

Thaw REPLI-g Mini Kit DNA Polymerase on ice, other reagents room temperature, vortex and then spin briefly.

Step 7 Preparation of the MasterMix

After addition of nuclease-free water and REPLI-g Mini Reaction Buffer, briefly vortex and centrifuge mixture before addition of DNA polymerase (preparation on ice and immediate use after addition of DNA polymerase).

Step 8 Add 40 µl or **30 µl of the master mix** to 10 µl or **20 µl of denatured DNA**.

Step 9 Incubate at 30° for 10-16 hours

Step 10 Inactive DNA Polymerase by heating the sample for 3 minutes at 65°

Step 11 If performing PCR, dilute the amplified DNA (1:20, 2µl amplified DNA, 38µl TE) and use 3 µl of diluted DNA for each PCR

Step 12 Store amplified DNA at 4° for short-term storage or -20° for long-term storage

Step 11 and 12 was not applicable for our protocol.

Purification of REPLI-g amplified DNA using Agencourt AMPure XP magnetic beads:

Step 1 After REPLI-g reaction, equilibrate REPLI-g amplified DNA to room temperature (15-25°) for no longer than 10 minutes

Step 2 Adjust the volume to 60 µl by adding the appropriate volume of TE buffer (if necessary)

Step 3 Vortex Agencourt AMPure XP bottle to resuspend any magnetic particles and add 108 µl of resuspended Agencourt AMPure XP beads to 60 µl of REPLI-g amplified DNA

The sample to beads ratio was 1:1 according to Oyola SO, et al. (1)

Step 4 Mix thoroughly by pipetting up and down (> 10x)

Step 5 Incubate at room temperature for 5 minutes to allow binding of DNA to Agencourt AMPure XP beads

Step 6 Place tube into MPC for 2 minutes to separate beads from solution (extend the time until solution becomes clear)

Step 7 Remove cleared supernatant with a pipet and discard (while the tube is situated on MPC – avoid pipetting of magnetic beads)

Step 8 Add 200 µl of ethanol (70%) to each tube comprising separated magnetic beads and incubate for 30 seconds at room temperature (while the tube is situated on MPC – avoid pipetting of magnetic beads)

Step 9 Carefully remove the cleared supernatant

Step 10 Repeat steps 8-9 twice (ensure that ethanol is completely removed from the bottom)

Step 11 Incubate microcentrifuge tube for 5 minutes at room temperature

Step 12 Remove tubes from the MPC, add 60 µl of 1 x TE buffer (pH 8.0) to dissolve REPLI-g amplified DNA, and pipet 10 times to mix (mix carefully to avoid shearing of REPLI-g amplified DNA)

Step 13 Place reaction onto the MPC for 1 minute to separate the beads from the solution

Step 14 Transfer the eluate to a new tube

Step 15 If not being used directly, store the amplified DNA/cDNA at -15 to -30° until required for downstream applications

1. Oyola SO, Manske M, Campino S, Claessens A, Hamilton WL, Kekre M, et al. Optimized whole-genome amplification strategy for extremely AT-biased template. *DNA Res* 2014;21:661–671.
