# Supplementary material for: The composition and functional protein subsystems of the human nasal microbiome in granulomatosis with polyangiitis: a pilot study
Source: Microbiome. 2019 Oct 22;7:137. doi: 10.1186/s40168-019-0753-z (PMC6806544; doi:10.1186/s40168-019-0753-z)
Supplement: Supplementary file 12 — Additional file 12. Bioinformatics Analysis (DOCX 45 kb) [file 40168_2019_753_MOESM12_ESM.docx]

**Supplementary Bioinformatics Analysis**

**Content:**

**2.4 Bacterial 16S rRNA gene library preparation and Illumina MiSeq sequencing 1**

**2.6 Bioinformatics 2**

Bacterial 16S rRNA marker gene analysis 2

Oligotyping and species identification 3

Contamination detection in bacterial 16S samples 4

Shotgun metagenomic sequence analysis 5

*Staphylococcus* isolates sequence analysis 6

Functional analysis of Shot gun metagenomic sequences 6

**2.7 Statistical and visual data analysis 7**

Permanova 7

Non-metric multidimensional scaling (NMDS) 7

**2.8 Analysis of disease duration with bacterial profile 8**

**References 9**

**2.4 Bacterial 16S rRNA gene library preparation and Illumina MiSeq sequencing**

The bacterial 16S rRNA V1V2 variable region of extracted DNA was amplified with Illumina adapter and indexed PCR primers using a dual-index sequencing strategy to target the bacterial 16S rRNA gene (1). Each PCR was done in triplicate in a total reaction volume of 25 μl together with 200μM deoxynucleotide triphosphates (dNTPs), 0.5μM V1 forward primers (7f 5' AATGATACGGCGACCACCGAGATCTACAC-XXXXXXXX-acactctttccctacacgacgctcttccgatct-NNNN-AGMGTTYGATYMTGGCTCAG3'), 0.5μM V2 reverse primer (r356 5' –CAAGCAGAAGACGGCATACGAGAT-XXXXXXXX-gtgactggagttcagacgtgtgctcttccgatct-NNNN-GCTGCCTCCCGTAGGAGT- 3'), and 0.25μl Q5 Taq enzyme. The Illumina adapter primer sequence is built of <Illumina adapter> < 8 bp index sequences> <binding side for Illumina sequencing primer> <4 maximally degenerated bases> ("NNNN") to maximize diversity during the first four bases of the run and <PCR target sequence>. Cycling conditions were as follows: Denaturation at 98°C for 2 minutes, followed by 30 cycles of amplification (denaturation 98°C for 30 seconds, annealing 50°C for 30 seconds, extension 72°C for 90 seconds) and a final extension at 72°C for 5 minutes. All primers were purchased from Eurofins (Ebersberg, Germany). Triplicate PCR reactions were pooled and purified with 75μl AMPure XP beads (Agencourt Bioscience) according to Illumina's 16S metagenomic sequencing library preparation protocol, pages 8-9 (Part # 15044223 Rev. B,1127/2013 (2).

DNA concentration were quantified using the Qubit 2.0 fluorometer and high sensitivity DNA reagents (Invitrogen). Samples were pooled in equimolar concentration and gel purified using The Wizard® SV Gel and PCR Clean-Up System (Promega). The library size was confirmed on a Tape station (Agilent Technologies) and then MiSeq sequenced using the 600 cycle MiSeq reagent kit V3, which enables 300 base pair end sequencing. The library was sequenced at the Wellcome Sanger Institute (Cambridge, United Kingdom). 104 samples including negative and positive controls were sequenced in one library.

**2.6 Bioinformatics**

Bacterial 16S rRNA maker gene analysis

The forward and reverse fastq files of each sample were processed according to the MOTHUR MiSeq SOP with some modifications (MOTHUR wiki at <http://www.mothur.org/wiki/MiSeq_SOP>). The "make.contigs" command was used with no extra parameters. The assembled contigs were taken out from the MOTHUR pipeline and the four poly NNNNs present in the adapter/primer sequences were removed using the "-trim_left 4" and "-trim_right 4" parameters in the PRINSEQ program (3). The PRINSEQ trimmed sequences were used for the first "screen.seqs" command to remove ambiguous sequences (maxambig=0) and sequences containing homopolymers longer than 8 bp (maxhomop=8). The quality-screened sequences were aligned using the Silva bacterial database "silva.nr_v123.align" with flip parameter set to true. Any sequences outside the expected alignment coordinates were further removed using the "screen.seqs" command. The alignment coordinates were set with "optimize=start-end, criteria=90". In addition, any sequences longer than 400 bp were remove with "maxlength=400". The correct aligned sequences were filtered using the "filter.seqs" command with "vertical=T" and "trump=.". The subsequent filtered sequences were de-noised by allowing three mismatches in the "pre.clustering" step and chimeras were removed using Uchime with the dereplicate option set to "true". The chimera free sequences were classified using the Silva reference database "silva.nr_v123.align" and the Silva taxonomy database "silva.nr_v123.tax" and a cut off value of 80%. Chloroplast, Mitochondria, unknown, Archaea, and Eukaryota sequences were removed. The high quality, chimera free, and correct classified sequences were normalized using the "sub.sample" command. Each sample was normalized to 10114 reads. This removed 35 samples. Ten important samples (mainly longitudinal samples with reads below 10114 but above 1000 reads were added back in to the datasets. In addition, the PCR negative control with only 24 sequences was added back in as well. Off the total 79 samples, three positive controls were taken out leaving 76 samples for oligotyping and species identification. Two positive controls (Staphylococcus aureus DNA samples) were used for sequence error calculation and one positive control was a genomic DNA from microbial mock community B, v5.1H, from Beiresources (ATCC USA). The overall sequence error rates were 0.00884% and 0.00482%, respectively.

Oligotyping and species identification

Oligotyping was used for clustering the high quality filtered fasta sequences from the MOTHUR pipeline. Oligotyping is a computational method to investigate the diversity of closely related by distinct bacterial organisms in final operational taxonomic units identified in environmental data sets through 16S riobosomal RNA gene data by the canonical approaches. For oligotyping we used the “Minimum Entropy Decomposition” (MED) option for sensitive partitioning of high-throughput marker gene sequences from the oligotyping pipeline (4). The normalized high quality fasta and name file from MOTHUR were renamed by appending the group name to the sequence name, using the "rename.seqs" command. Then, a redundant renamed-fasta file was generated using the "deunique.seqs" command, which creates a redundant fasta file from a fasta and name file. The redundant fasta file was subsequently used for oligotyping using the unsupervised "Minimum Entropy Decomposition" (MED) for sensitive partitioning of high-throughput marker gene sequences (4). The command line was "decompose fasta.file --gen-html –g –t -" using default parameters accept the –t character which was set to a dash "-" character. The dash character was used in MOTHUR "rename.seqs" command to separate the sample name from the unique info in the defline of the sequence name.

The default MED parameters calculated a minimum substantive abundance of an oligotype (-M) of 139. This default setting removed 4.72% of sequences (32,868/694,885) and generated 137 final nodes (oligotypes). MED analysis was repeated with a 10 fold lower minimum substantive abundance of an oligotype (-M 14, 137 final nodes divided by 10). This refined setting removed 1.59% of sequences (11,0871 / 694,885) and generated 688 oligotypes (OTPs). The 688 oligotypes could be grouped into 378 unique (non-redundant) oligotypes. Analysis was continued with oligotypes with a minimum abundance of 0.01% which retained 152 OTP species. These 152 OTP species covered 97.06% of all sequence reads. The 152 OTP matrix table was used for contamination detection.

The node representative sequence of each oligotype (OTP) was used for species profiling using the ARB analysis - A Software Environment for Sequence Data (version 5.5-org-9167) (5). For ARB analysis we used a customized version of the SILVA SSU Ref database (NR99, release 123) that was generated by removing environmental and uncultured taxa.

Contamination detection in Bacterial 16S samples

We followed a very stringent in-house pipeline to remove environmental and laboratory contaminants. By using various guidelines outlined in published work by Salter, et al, 2014 (6) we mitigated the effects of exogenous kit associated DNA contamination. The methods we used to reduce the effects of contamination included using sequencing appropriate controls, recording the kit from which samples were extracted, reducing contamination as much as possible during collection and extraction by using sterile materials and applying general aseptic techniques, and randomising sample handling as much as logistically possible. Once the sequencing was performed, contaminant OTPs were identified using the negative controls, or association with extraction kit, batch or sequencing run. In a second step we used Spearman rho’s correlation coefficient analysis to correlate obvious contaminants (e.g. Stenotrophomonas, Undibacterium, Herbaspirillum, and Sphingomonas) with suspicious contaminants. In a third step we removed low-abundance species reported in environmental habitats and as not cultured from human specimens. Out of the 152 high quality OTP species, 88 were considered to be contaminants. 64 high quality and contamination filtered OTP species were used final analysis.

Shotgun metagenomic sequence analysis

Human sequences were removed by aligning the raw reads against the human genome reference GRCh38 using the bowtie aligner as part of the KneadData software (v 0.6.1). Trimmomatic software (v 0.33-1) was used to remove Illumina adapters, to remove nine base pairs from the 5’ end of the reads, to conduct a quality filtering with a minimum score of 20 over a sliding window of 4, and for a minimum length filtering of 30 base pairs. The Trimmomatic filtering was part of the KneadData software (http://huttenhower.sph.harvard.edu/kneaddata). The high quality filtered reads were then used for contigs assembly using SPAdes (v 3.11.1) with meta option (8). SPAdes scaffolds were used for DIAMOND (v 0.8.4.66) blastx search against the NCBI non-redundant nucleotide database using standard settings (e value 0.001), except the maximum number of target sequences per query to keep for alignments was set to 1 (-k 1), and the number of chunks for processing the seed index was set to 1 for maximum performance (9). DIAMOND blastx files were subsequently used for MEGAN (v 6.10.5) analysis (10). MEGAN places sequence reads into the taxonomy by assigning each read to a taxon at some level in the NCBI taxonomic tree (taxonomic tree used in v 6.10.5 has 1,601,132 nodes), based on their hits to known sequences using a lowest common ancestor (LCA) assignment algorithm. We used the default LCA parameters.

*Staphylococcus* isolates sequence analysis

Sequence data were assembled using an in-house pipeline (11). Briefly, for each isolate the sequence reads were used to create multiple assemblies using VelvetOptimiser v2.2.5 and Velvet v1.2 (12). The assemblies were improved by scaffolding the best N50 and contigs using SSPACE (13) and sequence gaps filled using GapFiller (14). Multilocus sequence types (MLST) were determined from the assemblies using MLST check (https://github.com/sanger-pathogens/mlst_check), which was used to compare the assembled genomes against the MLST database for *S. aureus* (<http://pubmlst.org/saureus/>).

Functional analysis of Shot gun metagenomic sequences

For functional analysis of shotgun metagenomic sequence data we used the functional classification systems of MEGAN using SEED protein subsystem classification. The acc2seed-May2015XX.abin file was uploaded into MEGAN during the Diamond tblastx analysis as described above in “Shotgun metagenomic sequence analysis”. The user manual of MEGAN V6.13.5 by Daniel H. Huson, January 7, 2019, describes the SEED classification of gene function as a collection of biologically defined subsystems. The MEGAN program will attempt to map each read onto a gene that has a known functional role. The SEED functional role from the level two classification was then analysed for significant involved SEED function using non-parametric Kruskal-Wallis test. Significant functions were then further analysed in GraphPad Prism 7.0E for Mac OS X and Dunn’s multiple comparisons test. The mean abundance with standard deviation of statistically implicated SEED function in the five sample groups were then plotted as a scatter dot plot.

For the association between functional genes and bacterial species we used a heat map annotation approach. In the heat map analysis, we focused on the most abundant species, thus the contamination of the SEED functional protein subsystems is neglectable.

**2.7 Statistical and visual data analysis**

Permanova

For the Permanova test we used the statistically package PAST3 version 3.09 (15) using the Bray-Curtis distance measure and 9999 permutations . The Permanova test is better than the ANOVA/MANOVA test because it does not need normal distributed data and Euclidean distance but works with any distance measure and uses permutations to make it distribution free (16). The Permanova test generates a p-value and a F-statistic.

Spearman’s rho coefficient analyses were performed with PAST3 to identify patterns of association of bacterial OTP species with particular sample groups.

Non-metric multidimensional scaling (NMDS) and Correspondence analysis (CA)

The goal of non-metric multidimensional scaling (NMDS) is to represent the original position of data (samples) in multidimensional space as accurately as possible using a reduced number of dimensions that can be easily plotted and visualized.

NMDS is an indirect gradient analysis approach which produces an ordination based on a distance or dissimilarity matrix. NMDS is a rank-based approach, thus it attempts to represent as closely as possible, the pairwise dissimilarity between objects in a low-dimensional space. To run the NMDS, we used the function metaMDS from the VEGAN package in R (17,18).

In addition to NMDS we also used Correspondence analysis (CA), which again is an indirect gradient analysis based on a multivariate statistical technique similar to principal component analysis, it provides a means of displaying or summarising a set of data in two-dimensional graphical form. CA was plotted using PAST3. Data for CA were transformed to relative proportional abundance in Microsoft Excel for Mac version 15.41.

**2.8 Analysis of disease duration with bacterial 16S rRNA dataset and *Staphylococcus* shot gun metagenomic sequence data**

We used a heatmap analysis with annotation for disease duration and patient group information to identify any association between disease duration and the microbiome. The heatmap was generated with the R package ‘Heatplus’ version 2.28.0. For the bacterial 16S dataset we included the top 28 species with a minimum abundance of 0.5% in at least one sample. We set the “cuth” parameter in the heatmap as such that the clustering resulted in three clusters. The Beta diversity (microbial differences between the three cluster) was tested using PERMANOVA test as described in section 2.7. To compare the disease duration between the three cluster we used the non-parametric Kruskal-Wallis test using Dunn’s multiple comparison test in Graph Prism 7 for Mac. Since we found some differences in disease duration in the 16S dataset, we reported the mean, minimum-maximum and standard deviation of the duration of disease between the three clusters. For the *Staphylococcus* shot gun metagenomic dataset we included the top 18 *Staphylococcus* species with a minimum abundance of 0.5% in at least one sample. The “cuth” parameter was set as such that the clustering resulted in four clusters. The same tests were further used as described above.

References

1. Kozich JJ, Westcott SL, Baxter NT, Highlander SK, Schloss PD. Development of a dual-index sequencing strategy and curation pipeline for analyzing amplicon sequence data on the miseq illumina sequencing platform. *Appl Environ Microbiol* 2013;79:5112–5120.

2. Anon. 16S Metagenomic Sequencing Library Preparation. Available at: http://support.illumina.com/content/dam/illumina-support/documents/documentation/chemistry_documentation/16s/16s-metagenomic-library-prep-guide-15044223-b.pdf. Accessed February 22, 2018.

3. Schmieder R, Edwards R. Quality control and preprocessing of metagenomic datasets. *Bioinformatics* 2011;27:863–864.

4. A Murat Eren, Hilary G Morrison, Pamela J Lescault, Julie Reveillaud JHV and MLS. Minimum entropy decomposition: Unsupervised oligotyping for sensitive partitioning of high-throughput marker gene sequences. *ISME J* 2015;9:968–979.

5. Ludwig W, Strunk O, Westram R, Richter L, Meier H, Yadhukumar A, et al. ARB: A software environment for sequence data. *Nucleic Acids Res* 2004;32:1363–1371.

6. Salter S, Cox MJ, EM T, Calus S, Cookson W, Moffatt M, et al. Reagent and laboratory contamination can critically impact sequence-based microbiome analyses. *BMC Bioinformatics* 2014;12:87.

7. Marks M, Mitjà O, Vestergaard LS, Pillay A, Knauf S, Chen C-Y, et al. Challenges and key research questions for yaws eradication. *Lancet Infect Dis* 2015;15:1220–1225.

8. Bankevich A, Nurk S, Antipov D, Gurevich AA, Dvorkin M, Kulikov AS, et al. SPAdes: A New Genome Assembly Algorithm and Its Applications to Single-Cell Sequencing. *J Comput Biol* 2012;19:455–477.

9. Buchfink B, Xie C, Huson DH. Fast and sensitive protein alignment using DIAMOND. *Nat Methods* 2015;12:59–60. Available at: http://www.nature.com/doifinder/10.1038/nmeth.3176%5Cnhttp://dx.doi.org/10.1038/nmeth.3176%5Cnhttp://www.nature.com/doifinder/10.1038/nmeth.3176%5Cnhttp://www.ncbi.nlm.nih.gov/pubmed/25402007.

10. Huson DH, Beier S, Flade I, Górska A, El-Hadidi M, Mitra S, et al. MEGAN Community Edition - Interactive Exploration and Analysis of Large-Scale Microbiome Sequencing Data. *PLoS Comput Biol* 2016;12.

11. Parkhill J, Quail MA, Hunt M, Silva N De, Keane JA, Page AJ, et al. Robust high-throughput prokaryote de novo assembly and improvement pipeline for Illumina data. *Microb Genomics* 2016;2. Available at: http://www.microbiologyresearch.org/content/journal/mgen/10.1099/mgen.0.000083.

12. Zerbino DR, Birney E. Velvet: Algorithms for de novo short read assembly using de Bruijn graphs. *Genome Res* 2008;18:821–829.

13. Boetzer M, Henkel C V., Jansen HJ, Butler D, Pirovano W. Scaffolding pre-assembled contigs using SSPACE. *Bioinformatics* 2011;27:578–579.

14. Boetzer M, Pirovano W. Toward almost closed genomes with GapFiller. *Genome Biol* 2012;13.

15. Hammer Ø, Harper DAT, Ryan PD. PAST: Paleontological statistics sofware package for education and data analysis. *Palaeontol Electron* 2001;4:1–9. Available at: http://books.google.com/books?hl=pt-BR&lr=&id=KECV-qSRSUYC&pgis=1.

16. Anderson MJ, Walsh DCI. PERMANOVA, ANOSIM, and the Mantel test in the face of heterogeneous dispersions: What null hypothesis are you testing? *Ecol Monogr* 2013;83:557–574.

17. Oksanen J. Vegan: an introduction to ordination. *Management* 2008;1:1–10. Available at: http://doi.acm.org/10.1145/2037556.2037605%5Cnftp://ftp3.ie.freebsd.org/pub/cran.r-project.org/web/packages/vegan/vignettes/intro-vegan.pdf.

18. Oksanen J, Blanchet FG, Friendly M, Kindt R, Legendre P, McGlinn D, et al. *vegan: Community Ecology Package*.; 2016.
